# TREM-1 Deficiency Can Attenuate Disease Severity without Affecting Pathogen Clearance

**DOI:** 10.1371/journal.ppat.1003900

**Published:** 2014-01-16

**Authors:** Benjamin Weber, Steffen Schuster, Daniel Zysset, Silvia Rihs, Nina Dickgreber, Christian Schürch, Carsten Riether, Mark Siegrist, Christoph Schneider, Helga Pawelski, Ursina Gurzeler, Pascal Ziltener, Vera Genitsch, Fabienne Tacchini-Cottier, Adrian Ochsenbein, Willy Hofstetter, Manfred Kopf, Thomas Kaufmann, Annette Oxenius, Walter Reith, Leslie Saurer, Christoph Mueller

**Affiliations:** 1 Division of Experimental Pathology, Institute of Pathology, University of Bern, Bern, Switzerland; 2 Department of Biochemistry, University of Lausanne, Epalinges, Switzerland; 3 Department of Clinical Research, University of Bern, Bern, Switzerland; 4 Institute of Molecular Health Sciences, ETH Zurich, Zurich, Switzerland; 5 Institute of Pharmacology, University of Bern, Bern, Switzerland; 6 Institute of Microbiology, ETH Zurich, Zurich, Switzerland; 7 Department of Medical Oncology, University of Bern, Bern, Switzerland; 8 Department of Pathology and Immunology, Centre Medical Universitaire, Geneva, Switzerland; University of California, Los Angeles, United States of America

## Abstract

Triggering receptor expressed on myeloid cells-1 (TREM-1) is a potent amplifier of pro-inflammatory innate immune reactions. While TREM-1-amplified responses likely aid an improved detection and elimination of pathogens, excessive production of cytokines and oxygen radicals can also severely harm the host. Studies addressing the pathogenic role of TREM-1 during endotoxin-induced shock or microbial sepsis have so far mostly relied on the administration of TREM-1 fusion proteins or peptides representing part of the extracellular domain of TREM-1. However, binding of these agents to the yet unidentified TREM-1 ligand could also impact signaling through alternative receptors. More importantly, controversial results have been obtained regarding the requirement of TREM-1 for microbial control. To unambiguously investigate the role of TREM-1 in homeostasis and disease, we have generated mice deficient in *Trem1*. *Trem1^−/−^* mice are viable, fertile and show no altered hematopoietic compartment. In CD4^+^ T cell- and dextran sodium sulfate-induced models of colitis, *Trem1^−/−^* mice displayed significantly attenuated disease that was associated with reduced inflammatory infiltrates and diminished expression of pro-inflammatory cytokines. *Trem1^−/−^* mice also exhibited reduced neutrophilic infiltration and decreased lesion size upon infection with *Leishmania major*. Furthermore, reduced morbidity was observed for influenza virus-infected *Trem1^−/−^* mice. Importantly, while immune-associated pathologies were significantly reduced, *Trem1^−/−^* mice were equally capable of controlling infections with *L. major*, influenza virus, but also *Legionella pneumophila* as *Trem1^+/+^* controls. Our results not only demonstrate an unanticipated pathogenic impact of TREM-1 during a viral and parasitic infection, but also indicate that therapeutic blocking of TREM-1 in distinct inflammatory disorders holds considerable promise by blunting excessive inflammation while preserving the capacity for microbial control.

## Author Summary

Triggering receptor expressed on myeloid cells-1 (TREM-1) is an immune receptor expressed by myeloid cells that has the capacity to augment pro-inflammatory responses in the context of a microbial infection. While a TREM-1-amplified response likely serves the efficient clearance of pathogens, it also bears the potential to cause substantial tissue damage or even death. Hence, TREM-1 appears a possible therapeutic target for tempering deleterious host-pathogen interactions. However, in models of bacterial sepsis controversial findings have been obtained regarding the requirement of TREM-1 for bacterial control - depending on the overall degree of the TREM-1 blockade that was achieved. In order to conclusively investigate harmful versus essential functions of TREM-1 *in vivo*, we have generated mice deficient in *Trem1*. *Trem1^−/−^* mice were subjected to experimentally-induced intestinal inflammation (as a model of a non-infectious, yet microbial-driven disease) and also analysed following infections with *Leishmania major*, *influenza virus* and *Legionella pneumophila*. Across all models analysed, *Trem1^−/−^* mice showed substantially reduced immune-associated disease. We thus describe a previously unanticipated pathogenic role for TREM-1 also during a parasitic and viral infection. Importantly, our data suggest that in certain diseases microbial control can be achieved in the context of blunted inflammation in the absence of TREM-1.

## Introduction

Innate immune cells express several cell surface receptors and intracellular sensing molecules that allow for autonomous recognition of pathogen- and danger-associated molecular patterns (PAMPs and DAMPs) and initiation of pro-inflammatory anti-microbial reponses. Toll-like receptors (TLR) and nucleotide-binding oligomerization domain (NOD)-like receptors, which recognize a diverse group of highly conserved microbial structures, represent only two examples of large innate immune receptor families with activating functions. Over the last decade, an additional family of evolutionary conserved innate immune receptors has been identified and characterized, the so-called triggering receptors expressed on myeloid cells (TREMs). TREMs belong to the immunoglobulin (Ig) superfamily of receptors and contain both inhibitory and activating receptors [1], [2], [3]. In contrast to the fairly ubiquitously expressed TLRs and NOD-like receptors, expression of TREMs is restricted to cells of the myeloid lineage [4]. Moreover, based on their capacity to integrate and potently modulate TLR- and NOD-induced signals, TREMs appear to mainly act as fine-tuners rather than initiators of inflammatory responses [3], [5]. While TREM-1, TREM-2, TREM-3 (in the mouse) receptors [4], [6], and the TREM-1 like transcripts TLT-1 and TLT-2 have been described [7], [8], TREM-1 is the first identified and best characterized receptor of the TREM family with activating functions. TREM-1 consists of an ectodomain, composed of a single Ig V-type domain, a transmembrane region and a short cytoplasmic tail that recruits DAP12 for signaling [4]. TREM-1 is constitutively expressed on neutrophils and on subsets of monocytes and macrophages, and TREM-1 expression is further upregulated upon exposure of cells to microbial products [4]. Whereas crosslinking of TREM-1 with agonistic antibodies alone induces only modest cellular activation, TREM-1 potently synergizes with distinct TLR ligands for a substantial amplification of oxidative burst and production of pro-inflammatory mediators such as TNF, IL-1β, IL-6, IL-8, MCP-1 and Mip-1α [4], [9], [10].

*In vivo*, the role of TREM-1 has been mostly characterized in experimental models of endotoxin-induced shock or microbial sepsis where blockade of TREM-1 signaling conferred significant protection [9], [11], [12]. The detection of TREM-1 in inflammatory lesions caused by bacterial or fungal agents, but not in psoriasis or immune-mediated vasculitis [9], has further led to the general concept that TREM-1 is primarily involved in microbial diseases, particularly, since elevated levels of the serum soluble form of the shed TREM-1 surface receptor (sTREM-1) also appear to associate with bacterial infections in patients with pneumonia or suspected sepsis [13], [14].

However, increasing evidence is now emerging that TREM-1 may additionally play a role in non-infectious inflammatory conditions. Thus, expression of TREM-1 can also be induced by the non-microbial agent monosodium urate monohydrate crystals (MSU) or by hypoxic cell culture conditions *in vitro* [15], [16]. Augmented sTREM-1 levels have been reported for patients with rheumatoid arthritis, acute pancreatitis, chronic obstructive pulmonary disease and cardiac arrest [17], [18], [19], [20]. Furthermore, we have previously described an involvement of TREM-1 in human inflammatory bowel diseases (IBD) and in models of experimental colitis [21], [22], [23].

Investigations on the precise function of TREM-1 in distinct diseases have so far been complicated by the still unidentified ligand(s) for TREM-1. Putative ligands for TREM-1 have been described on the surface of human platelets and on murine granulocytes during experimental peritonitis and endotoxaemia [12], [24], [25]. In addition, necrotic cell lysates also appear to stimulate pro-inflammatory responses in a TREM-1-dependent manner, which may relate to association of TREM-1 with the High Mobility Group Box 1 (HMGB1) protein [26], [27]. Hence, it can be speculated that not only PAMPs but also DAMPs induce signaling *via* TREM-1 and that several ligands for TREM-1 may exist.

In the absence of clearly defined ligands for TREM-1, studies addressing the impact of TREM-1 in disease have so far mostly relied on the use of TREM-1/Ig fusion proteins or synthetic peptides mimicking part of the extracellular domain of TREM-1. Although by the use of these agents substantial protection from endotoxin-induced shock, microbial sepsis or experimental colitis could be conferred [9], [11], [12], [22], several aspects regarding the true biological role of TREM-1 remain unclear. First, considering the redundancy of innate immune receptor-ligand interactions, the possibility exists that in these previous studies not only signaling through TREM-1 but through additional, potentially more relevant receptors was prevented. Second, controversial findings have been obtained with respect to the impact of impaired TREM-1 signaling on microbial control [9], [28], [29], [30].

In order to investigate the role of TREM-1 in homeostasis and disease, we have generated a TREM-1-deficient (*Trem1^−/−^*) mouse by targeted deletion of exon 2. Here we show, employing distinct inflammation and infection models ranging from experimental colitis to infections with *Leishmania major*, influenza virus and *Legionella pneumophila*, that complete absence of TREM-1 significantly attenuates morbidity and immune-mediated pathologies while microbial control remains unimpaired. These findings not only demonstrate an unanticipated clear role for TREM-1 in chronic inflammatory disorders, parasitic and viral infections, but also illustrate the potential for a novel therapeutic intervention in various disease settings.

## Results

### Deletion of *Trem1* has no apparent impact under homeostatic conditions

**Figure 1:**
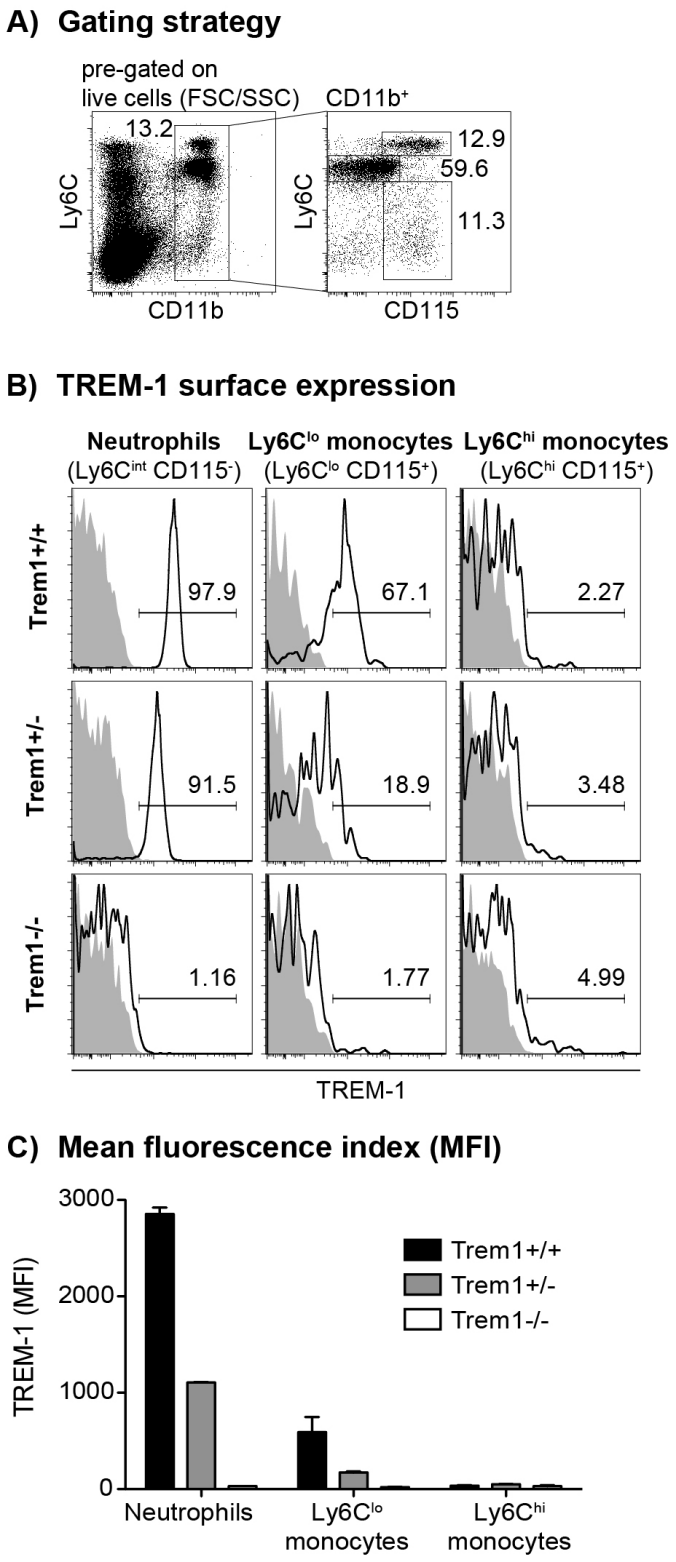
TREM-1 surface expression by peripheral blood myeloid cell subsets from wildtype versus *Trem1*-deficient mice. Peripheral blood cells obtained from wildtype (*Trem1^+/+^*) and *Trem1*-deficient (*Trem1^+/−^* and *Trem1^−/−^*) mice (n = 2 mice for each group) were stained for surface expression of TREM-1 and analysed by FACS. (A) Representative gating strategy to identify neutrophils and LyC6^lo^ and Ly6C^hi^ monocytes. (B) Representative histograms showing TREM-1 surface expression (lines) versus isotype controls (filled histograms). (C) Mean fluorescence intensity (MFI) of TREM-1 surface expression. Mean values of n = 2 mice analysed are shown with error bars indicating the range. Data are representative of several independent analyses.

**Figure 2:**
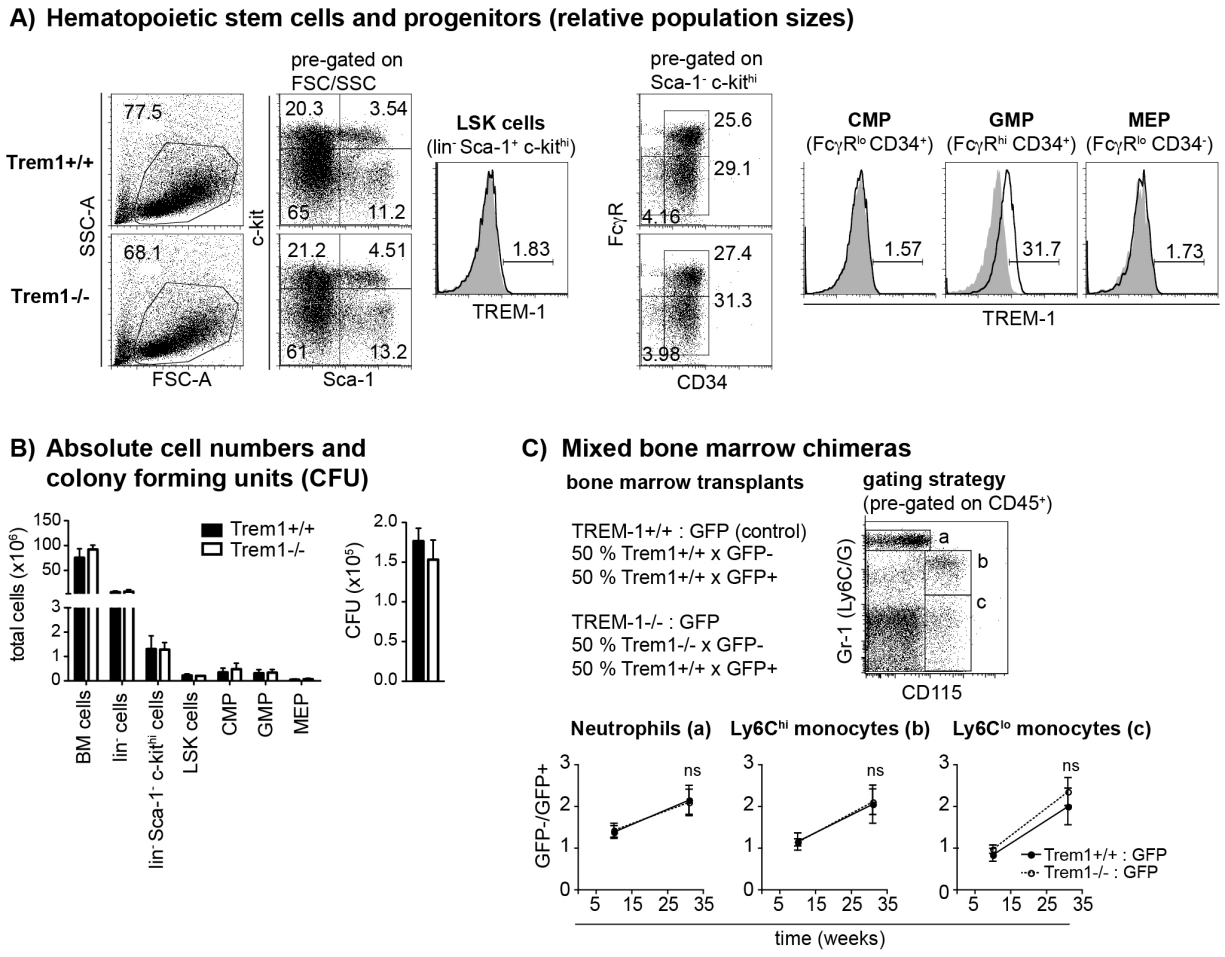
Unimpaired hematopoiesis in *Trem1^−/−^* mice. (A) Representative dot plots show the FACS-based identification of lineage-depleted (lin^−^) Sca1^+^ c-kit^hi^ (LSK) cells and lin^−^ Sca1^−^ c-Kit^hi^ myeloid progenitors in *Trem1^+/+^* (top panels) and *Trem1^−/−^* (bottom panels) bone marrow (BM) following lineage depletion and depletion of lymphoid progenitors by MACS. Common myeloid progenitors (CMP), granulocyte-macrophage progenitors (GMP) and megakaryocyte/erythrocyte progenitors (MEP) were further discriminated according to their expression of FcγR and CD34. Filled histograms show TREM-1 surface expression by LSK cells, CMP, GMP and MEP progenitors from *Trem1^−/−^* mice in comparison to *Trem1^+/+^* mice (lines). (B) Absolute cell numbers of total BM cells, lin^−^ BM cells, lin^−^ Sca1^−^ c-kit^hi^ myeloid progenitors, LSK cells, CMP, GMP and MEP and colony forming units (CFU) of hematopoietic precursors isolated from the BM of *Trem1^+/+^* and *Trem1^−/−^* mice were determined as described in the Materials and Methods section. Mean values of n = 2 mice analysed are shown with error bars indicating the range. (C) Mixed BM chimeras were generated by i.v. transfer of 1∶1 mixed *Trem1^+/+^ x GFP^+/+^* and *Trem1^−/−^ x GFP^−/−^* BM cells (white circles, dotted lines) into irradiated recipient mice. As control, and to account for potential interfering effects of the GFP expression, mixed BM from *Trem1^+/+^ x GFP^+/+^* and *Trem1^+/+^ x GFP^−/−^* mice (black circles and lines) was transferred into additional recipient mice. BM chimeras were analyzed after 10 and 31 weeks of chimerism. Neutrophils, Ly6C^hi^ and Ly6C^lo^ monocytes were identified in the peripheral blood according to the depicted gating strategy and the GFP^−^ : GFP^+^ cell ratio in the respective cell subsets was determined. Mean values of n = 4–5 mice analyzed per group are shown with error bars indicating the SEM. ns, no statistically significant difference. Data depicted in Figure 2 are representative of two independent experiments.

To account for potential embryonically lethal effects of a total deletion of the *Trem1* gene, a targeting vector was designed for conditional deletion of exon 2 (Fig. S1). Exon 2 encodes the extracellular domain of TREM-1 and also contains the putative ligand binding site [31]. Breeding of *Trem1^+/flox^* chimeric offspring mice with deleter mice that expressed Cre ubiquitously yielded viable *Trem1^+/−^ x Cre^+/−^* offspring. Moreover, interbreeding of *Trem1^+/−^* mice gave rise to *Trem1^−/−^* mice at the expected Mendelian frequencies, and *Trem1* ^−/−^ mice were equal in size, weight and fertility to littermate *Trem1^+/+^* controls. We thus continued to characterize *Trem1^−/−^* mice with a ubiquitously deleted *Trem1* gene by elementary flow cytometry analyses. Deletion of *Trem1* resulted in a gene-dose-dependent loss of TREM-1 surface expression by peripheral blood neutrophils and Ly6C^lo^ monocytes (Fig. 1). Accordingly, TREM-1 was still expressed at ∼2-fold reduced levels in *Trem1^+/−^* mice, while surface TREM-1 expression was absent on myeloid cells in *Trem1^−/−^* mice (Fig. 1). Absence of *Trem1* did not appear to affect the composition of various immune compartments, since numbers of distinct myeloid and lymphoid cell subsets isolated from the peripheral blood, bone marrow (BM) and spleen of *Trem1^−/−^* and *Trem1^+/+^* mice were identical (Fig. S2). However, to formally exclude a potential effect of TREM-1 on hematopoiesis, the BM of *Trem1^−/−^* and *Trem1^+/+^* mice was analysed in more depth with respect to hematopoietic stem cell and myeloid progenitor numbers following lineage depletion and depletion of lymphoid progenitors (Fig. 2). Stem cell-enriched cells were identified by their lineage^−^ (lin^−^) Sca-1^+^ c-kit^hi^ phenotype (LSK cells) while common myeloid progenitors (CMP), granulocyte/macrophage progenitors (GMP) and megakaryocyte/erythrocyte precursors (MEP) were discriminated within the Sca-1^−^ c-kit^hi^ population according to their differential expression of FcγR and CD34, respectively (Fig. 2a). Compared to *Trem1^+/+^* mice, *Trem1^−/−^* mice exhibited equal numbers of LSK cells, CMP, GMP and MEP (Fig. 2B). Moreover, similar numbers of colony forming units could be observed in lineage-depleted (lin^−^) BM cells isolated from *Trem1^−/−^* mice (Fig. 2B). Although these analyses indicated again that TREM-1 is unlikely to play a substantial role in hematopoietic processes, we were intrigued by the selective expression of surface TREM-1 by GMP, but not by CMP (Fig. 2A). As a final measure, we therefore established mixed bone marrow chimeras with either *Trem1^−/−^* (*x GFP^−/−^*) and *Trem1^+/+^ x GFP^+/+^* BM cells or, as a control, *Trem1^+/+^* (*x GFP^−/−^*) and *Trem1^+/+^ x GFP^+/+^* BM cells. Analysis of chimeric mice at 10 and 31 weeks post reconstitution and calculation of the respective ratios of GFP^−^ to GFP^+^ peripheral blood neutrophil, Ly6C^hi^ or Ly6C^lo^ monocyte numbers demonstrated an equal capacity of *Trem1^−/−^* BM to give rise to distinct myeloid subsets as *Trem1^+/+^* BM. Thus, while the potential role of TREM-1 expression by GMP still remains to be explored, deficiency in *Trem1* does not appear to affect hematopoietic processes under homeostatic conditions.

We next addressed whether absence of *Trem1* could affect other receptors that use DAP12 for signaling, either by the potential presence of increased levels of intracellularly available DAP12 or by the lack of counterregulatory signals conferred by TREM-1. Indeed, the hyperresponsive phenotype of DAP12-deficient macrophages is largely ascribed to a lack of inhibitory signals by TREM-2 which also employs DAP12 [32]. Due to the important role of TREM-2 in osteoclast formation and function [33], [34], we reasoned that lack of TREM-1 expression in *Trem1^−/−^* mice could possibly manifest in altered osteoclastogenesis. However, as determined by Xray and MicroCT analyses, no differences in bone density could be detected between *Trem1^−/−^* mice and their age- and sex-matched *Trem1^+/+^* controls (data not shown).

Taken together, these analyses revealed no apparent phenotype of *Trem1^−/−^* mice under homeostatic conditions.

### *Trem1^−/−^ x Rag2^−/−^* mice are largely protected from a CD4 T cell-induced colitis

**Figure 3:**
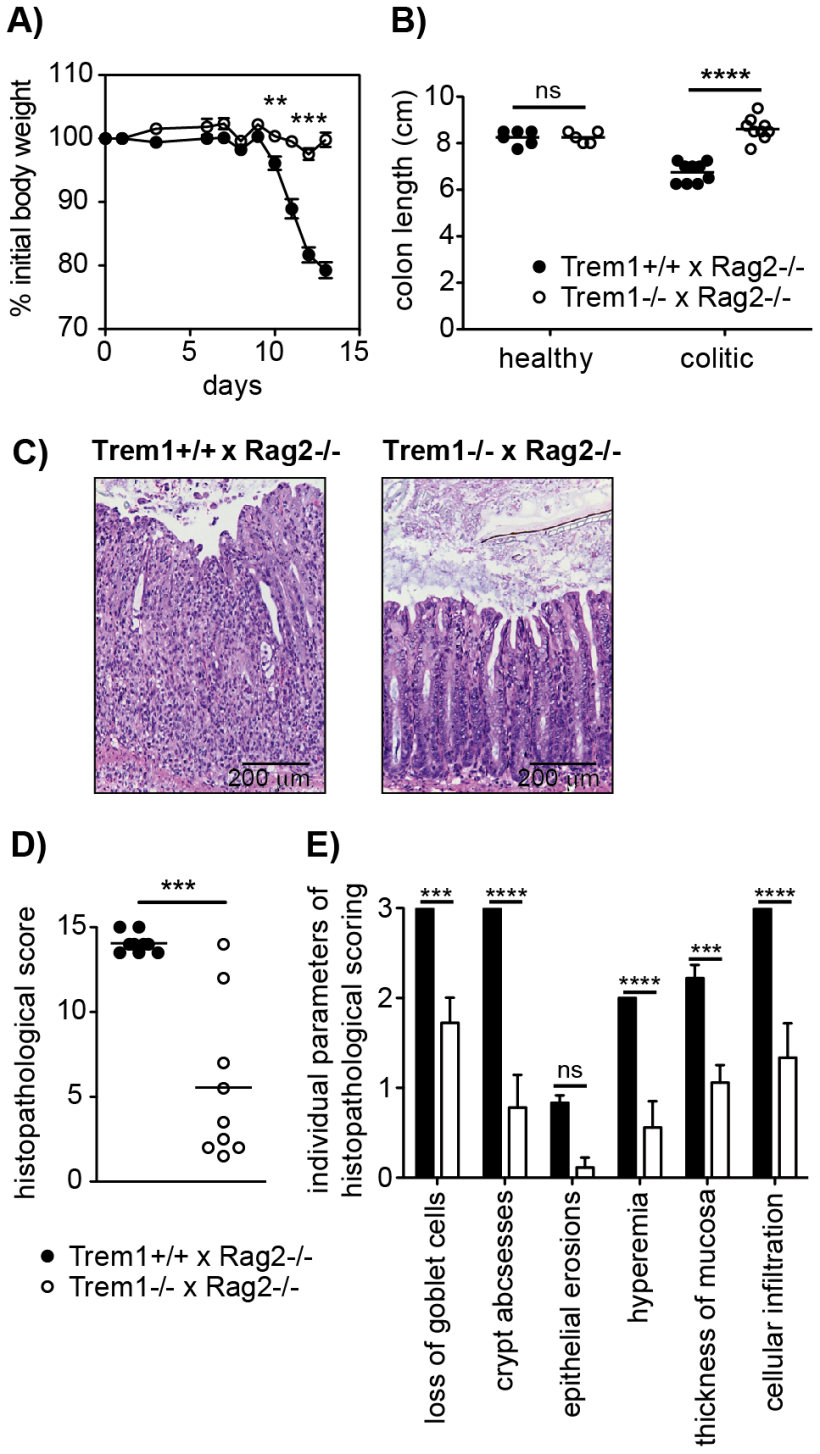
*Trem1^−/−^ x Rag2^−/−^* mice are protected from a CD4^+^ T cell-induced colitis. Colitis was induced in *Trem1^+/+^ x Rag2^−/−^* (filled circles) and *Trem1^−/−^ x Rag2^−/−^* mice (white circles) by i.p. injection of 2×10^5^ CD4^+^ CD45RB^hi^ T cells. (A) Weight loss relative to the initial body weight. Mean values of n = 9 mice analysed per group are shown with error bars indicating the SEM. (B) Colon lengths were determined in individual mice (symbols). Lines show mean values for each group of mice. (C) Representative H&E-stained colonic tissue sections of a *Trem1^+/+^ x Rag2^−/−^* (histopathological score: 14) and *Trem1^−/−^ x Rag2^−/−^* mouse (histopathological score: 2). (D) Total histopathological scores. Symbols show total scores for individual mice and lines indicate the mean value for each group of mice. Histopathological scores were determined for individual mice by a pathologist according to parameters defined in the Materials and Methods section. (E) Individual parameters of histopathological scoring. Columns show mean values for n = 9 mice analysed per group and error bars indicate the SEM. ****, p<0.0001; ***, p<0.001; **, p<0.01. One representative experiment out of three independent experiments is shown.

We have previously demonstrated a substantial accumulation of TREM-1 expressing macrophages in the inflamed, but not healthy intestinal mucosa of patients with IBD and of mice with experimental colitis [22], [23]. Hence, one of our major interests in the characterization of the *Trem1^−/−^* mouse was to unambigously investigate the role of *Trem1* in the pathogenesis of IBD. To this end, CD4^+^ CD25^−^ CD45RB^hi^ T cells were adoptively transferred into Helicobacter-positive *Trem1^+/+^* x *Rag2^−/−^* and *Trem1^−/−^ x Rag2^−/−^* recipient mice and animals were monitored regularly for clinical signs of colitis. Importantly, whereas *Trem1^+/+^ x Rag2^−/−^* mice lost ∼20% of their initial body weight at the end of the observation period, weight loss in *Trem1^−/−^ x Rag2^−/−^* mice was minimal and only transient (Fig. 3A). Furthermore, shortening of the colon was substantially attenuated in *Trem1^−/−^ x Rag2^−/−^* mice compared to controls (Fig. 3B). While some of the *Trem1^−/−^ x Rag2^−/−^* mice still exhibited moderate histopathological signs of intestinal inflammation, individual parameters of the histopathological scoring as well as the overall histopathological score were significantly reduced (Fig. 3C–E).

**Figure 4:**
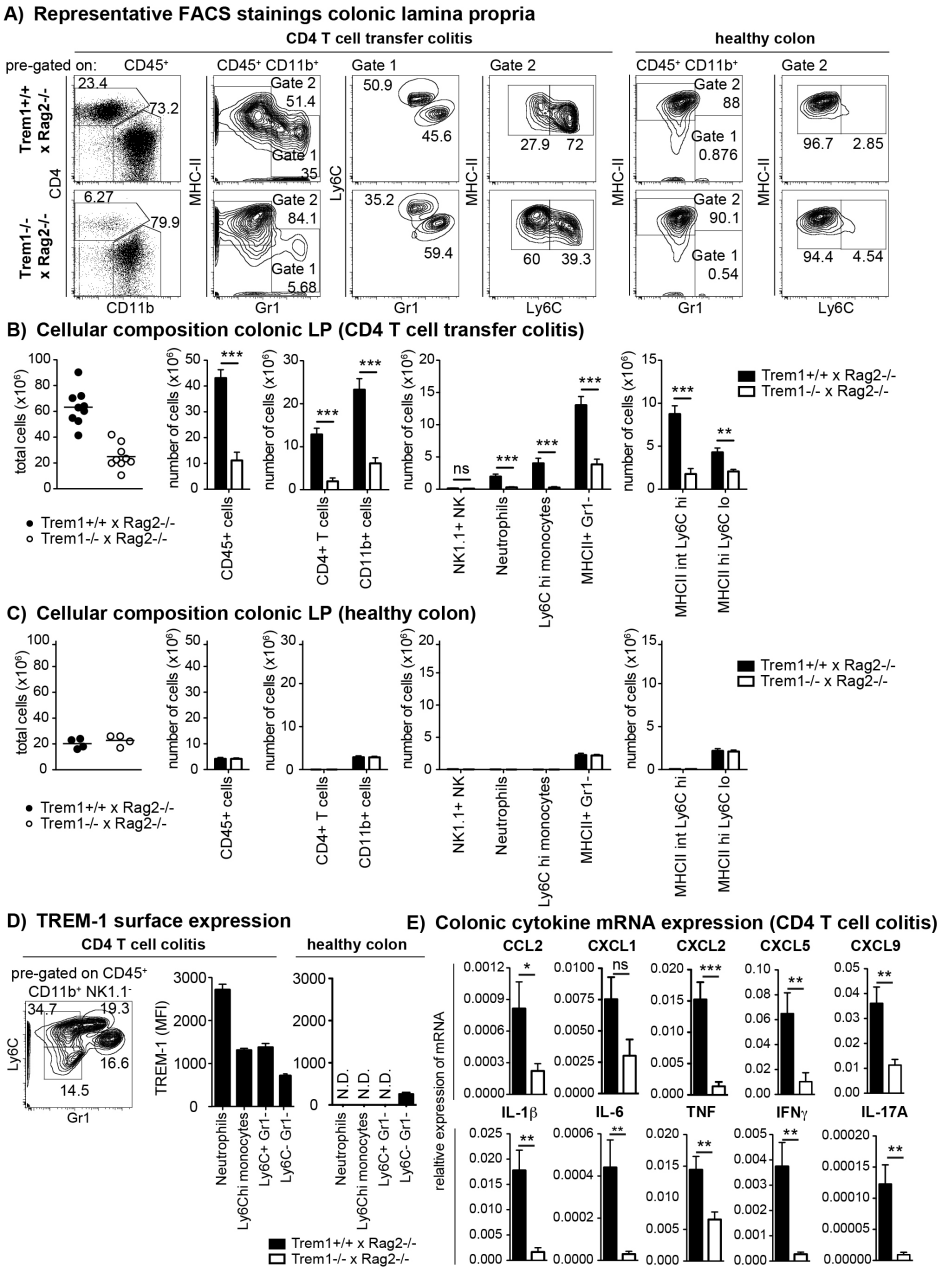
Upon colitis induction, *Trem1^−/−^ x Rag2^−/−^* mice exhibit substantially reduced inflammatory infiltrates and diminished expression of pro-inflammatory mediators. (A–C) Lamina propria cells were isolated from the colon of *Trem1^+/+^ x Rag2^−/−^* and *Trem1^−/−^ x Rag2^−/−^* mice 12–13 days post adoptive transfer of colitogenic CD4 T cells or from untransferred mice (healthy colons) and analysed by FACS. (A) After exclusion of doublets and dead cells, CD11b^+^ cells were discriminated from CD4^+^ T cells and further subgated into MHCII^lo^ Gr1^+^ (gate 1) and MHCII^hi^ Gr1^−^ (gate 2) cells. In gate 1, monocytes and neutrophils were identified according to their Ly6C^hi^ Gr1^int^ and Ly6C^int^ Gr1^hi^ phenotype, respectively. In gate 2, MHCII^+^ cells were further subdivided into two populations of MHCII^int^ Ly6C^hi^ and MHCII^hi^ Ly6C^lo^ cells. (B, C) Absolute numbers of total cells recovered from individual mice (symbols; lines indicate mean values per group) and mean values ± SEM for CD45^+^ cells, CD4^+^ T cells, CD11b^+^ cells and subsets defined within the CD11b^+^ gate as illustrated in (A). Per group, n = 9 mice adoptively transferred with CD4 T cells (B) and n = 4 untransferred (C) mice were analysed. (D) TREM-1 surface expression by neutrophils (Ly6C^int^ Gr1^hi^), monocytes (Ly6C^hi^ Gr1^int^) and CD11b^+^ Gr1^−^ Ly6C^+^ versus Ly6C^−^ subsets identified in the lamina propria (according to the gating strategy depicted in D) of colitic (n = 9) versus healthy (n = 4) *Trem1^+/+^ x Rag2^−/−^* mice. (E) Colonic tissues were assessed for the expression of pro-inflammatory mediators by qRT-PCR. Bars show mean values ± SEM for n = 9 mice. ***, p<0.001; **, p<0.01; *, p<0.05. N.D. = not determined due to insufficient cell numbers.

In order to gain insight in the potential underlying mechanism of the highly attenuated colitis in *Trem1^−/−^ x Rag2^−/−^* mice, colonic lamina propria cells that were isolated from both groups of mice in the absence of an adoptive CD4^+^ T cell transfer (healthy colon) or 12–13 days post colitis induction were analysed by FACS. As depicted in Fig. 4A, the colonic lamina propria of healthy *Trem1^+/+^ x Rag2^+/+^* mice and *Trem1^−/−^ x Rag2^−/−^* mice contained a similar proportion of CD11b^+^ MHCII^hi^ cells and Gr1^+^ cells were virtually absent. In contrast, Gr1^+^ cells, representing infiltrating Ly6C^hi^ Gr1^int^ monocytes and Ly6C^int^ Gr1^hi^ neutrophils, were readily detected in *Trem1^+/+^ x Rag2^+/+^* mice and *Trem1^−/−^ x Rag2^−/−^* mice at 12–13 days post colitis induction (Fig. 4A). Notably, the relative frequency of Gr1^+^ cells among CD45^+^ CD11b^+^ colonic LP cells was ∼5-fold lower in *Trem1^−/−^ x Rag2^−/−^* mice (Fig. 4A). In further contrast to the colonic LP of healthy mice, among LP MHCII^+^ Gr1^−^ cells of colitic mice two populations of MHCII^hi^ Ly6C^lo^ and MHCII^int^ Ly6C^hi^ cells could be discriminated, likely representing intestinal macrophages and monocytes in the process of differentiation (Fig. 4A) [35], [36]. Also within this gate of MHCII^+^ Gr1^−^ cells, substantial differences could be detected between the two groups of mice. Accordingly, the relative frequency of MHCII^int^ Ly6C^hi^ cells was increased in colitic *Trem1^+/+^ x Rag2^−/−^* mice whereas *Trem1^−/−^ x Rag2^−/−^* mice exhibited a larger proportion of MHCII^hi^ Ly6C^lo^ macrophages (Fig. 4A).

When colonic LP cells of n = 9 mice of both groups were systematically analysed at 12–13 days post colitis induction, substantially reduced numbers of various cell subsets could be seen in *Trem1^−/−^ x Rag2^−/−^* mice (Fig. 4B). Thus, *Trem1^−/−^ x Rag2^−/−^* mice not only exhibited reduced infiltrating CD4^+^ T cells but also significantly decreased numbers of neutrophils, Ly6C^hi^ monocytes and MHCII^int^ Ly6C^hi^ cells (Fig. 4B). These differences were not apparent for the colonic LP of healthy *Trem1^+/+^ x Rag2^−/−^* and *Trem1^−/−^ x Rag2^−/−^* mice which mainly contained MHCII^hi^ Ly6C^lo^ cells anyway (Fig. 4A and 4C).

To gain more insight which myeloid TREM-1-expressing cell subset could potentially be involved in driving intestinal inflammation in *Trem1^+/+^ x Rag2^−/−^* mice, TREM-1 surface expression was analysed on colonic LP neutrophils, Ly6C^hi^ monocytes as well as CD11b^+^ Ly6C^+^ Gr1^−^ and CD11b^+^ Ly6C^−^ Gr1^−^ cells. As reported previously [23], [37], in the healthy colonic LP TREM-1 expression was hardly detectable owing to the absence of infiltrating neutrophils and Ly6C^+^ cells (Fig. 4A and 4D). In colitic *Trem1^+/+^ x Rag2^−/−^* mice, TREM-1 expression was observed on neutrophils, Ly6C^hi^ monocytes and CD11b^+^ Ly6C^+^ Gr1^−^ cells (Fig. 4D). Moreover, CD11b^+^ Ly6C^−^ Gr1^−^ cells, likely representing intestinal macrophages, that were isolated from colitic *Trem1^+/+^ x Rag2^−/−^* mice exhibited a ∼3-fold upregulated expression of surface TREM-1 (Fig. 4D).

In line with the reduced infiltrating cell numbers, mRNA expression for various innate and adaptive pro-inflammatory chemokines and cytokines was significantly decreased in the lamina propria of *Trem1^−/−^ x Rag2^−/−^* compared to *Trem1^−/−^ x Rag2^−/−^* mice (Fig. 4E).

### Dextran sodium sulfate (DSS)-induced colitis is attenuated in *Trem1^−/−^* mice

**Figure 5:**
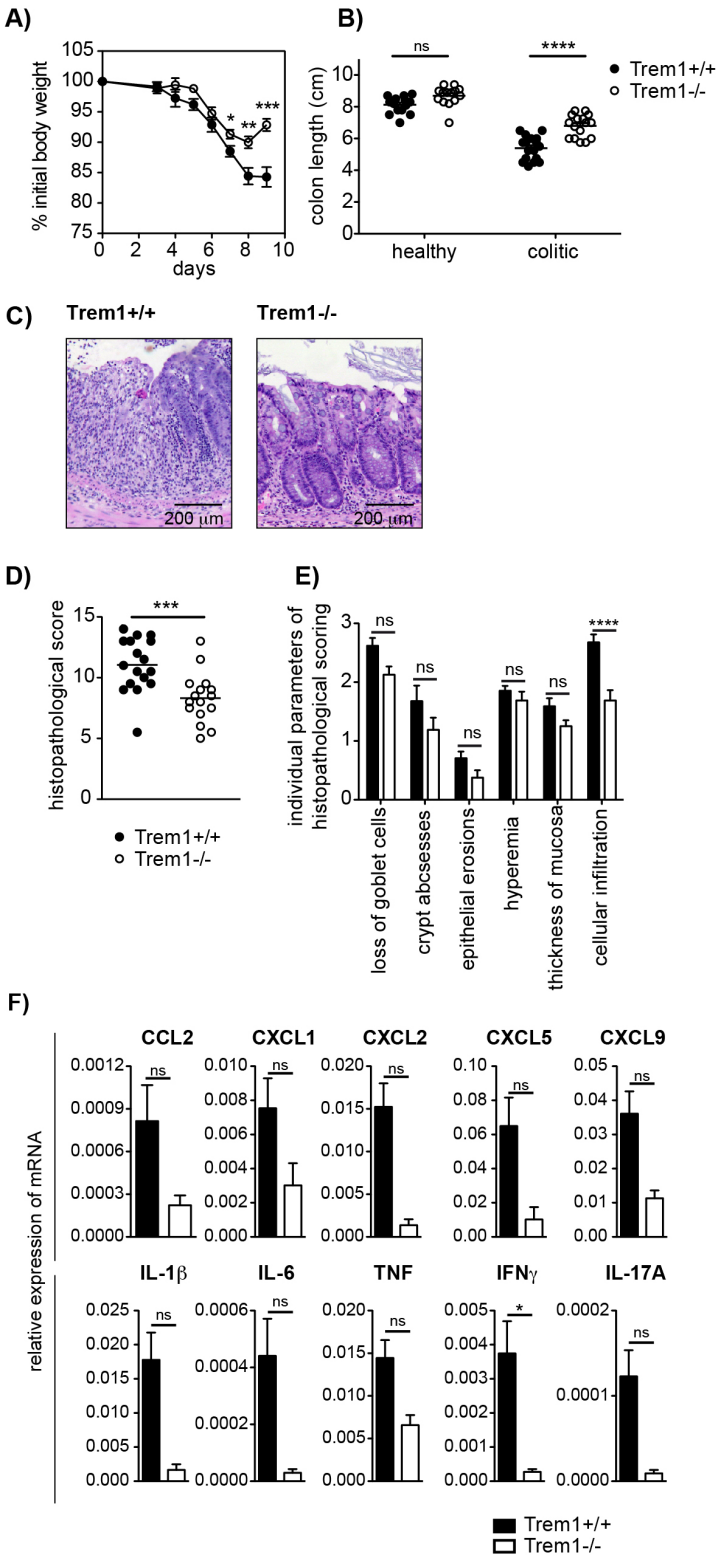
Attenuated dextran sodium-sulfate (DSS)-induced colitis in *Trem1^−/−^* mice. Colitis was induced in *Trem1^+/+^* and *Trem1^−/−^* mice by administration of 3% DSS in the drinking water for 5 days, followed by 4 days on regular tap water. (A) Weight loss relative to the initial body weight. Mean values of n = 17 (*Trem1^+/+^*) and n = 16 (*Trem1^−/−^*) mice are shown with error bars indicating the SEM. (B) Colon lengths were determined in individual DSS-treated and untreated control mice (symbols). Lines show mean values for each group of mice. (C) Representative H&E-stained colonic tissue sections of a *Trem1^+/+^* (histopathological score: 13) and *Trem1^−/−^* mouse (histopathological score: 5.5). (D) Total histopathological scores. Symbols show total scores for individual mice and lines indicate the mean value for each group of mice. Histopathological scores were determined for individual mice by a pathologist according to parameters defined in the Materials and Methods section. (E) Individual parameters of histopathological scoring. Columns show mean values and error bars indicate the SEM. (F) Colonic tissues were assessed for the expression of pro-inflammatory mediators by qRT-PCR. Bars show mean values ± SEM for n = 7 mice per group from one independent experiment. (A, B, D) Pooled data from three independent experiments are shown. ****, p<0.001; ***, p<0.001; *, p<0.05.

While protection from colitis in *Trem1^−/−^ x Rag2^−/−^* mice was associated with reduced expression of several pro-inflammatory mediators, previous data generated in our laboratory have demonstrated that TNF produced by nonlymphoid cells plays a non-redundant pathogenic role in the CD4^+^ T cell transfer model of colitis since *Tnf^−/−^ x Rag2^−/−^* mice are completely protected from colitis induction [38]. However, in acute models of intestinal inflammation such as the DSS-induced colitis, *Tnf^−/−^* mice exhibit aggrevated disease [39], [40], presumably, because early anti-microbial and repair responses following DSS-induced breaching of the epithelial barrier are fundamentally impaired. Due to the central function of TREM-1 in amplifying pro-inflammatory cytokine production and oxidative burst, we hypothesized that during acute intestinal inflammation complete absence of *Trem1* could also prove detrimental. Intriguingly, although upon administration of 3% DSS *Trem1^−/−^* mice initially lost weight to a similar extent as *Trem1^+/+^* mice, weight loss was considerably attenuated at 7 days post colitis induction and at 9 days *Trem1^−/−^* mice had already improved again (Fig. 5A). In *Trem1^−/−^* mice, shortening of the colon was markedly attenuated and total histopathological colitis scores were significantly decreased (Fig. 5B and 5C–E). Furthermore, analogous to the CD4 T cell-induced colitis (Fig. 4E), TREM-1 deficiency resulted in reduced colonic mRNA expression of several pro-inflammatory mediators (Fig. 5F). Hence, in contrast to *Tnf^−/−^* mice [39], [40], *Trem1^−/−^* mice still showed an adequate host response to DSS-induced epithelial injury while exhibiting reduced immune-mediated pathologies.

### Infection with *Leishmania major* leads to smaller inflammatory lesions with decreased neutrophilic cellular infiltrates in *Trem1^−/−^* mice

The observations made in the acute DSS model of colitis raised our interest whether *Trem1^−/−^* mice would also be able to control *bona fide* microbial infections, in particular, since maximal silencing of TREM-1 by a siRNA approach had proven deleterious in a fecal peritonitis model [29]. Since the rapid kinetics of this model hardly allowed to simultaneously look at beneficial effects of the *Trem1* deficiency on immune-mediated tissue damage or to assess potential adverse consequences for the priming of adaptive immune responses, we chose the *Leishmania major* infection model. Following infection with *L. major*, C57BL/6 mice develop local cutaneous lesions that spontaneously resolve within 4–8 weeks. Central to the resolution is the TNF-mediated control of the early inflammatory response or the clearance of neutrophils and the later IFNγ-mediated and Th1-driven elimination of the parasite by infected macrophages [41], [42].

**Figure 6:**
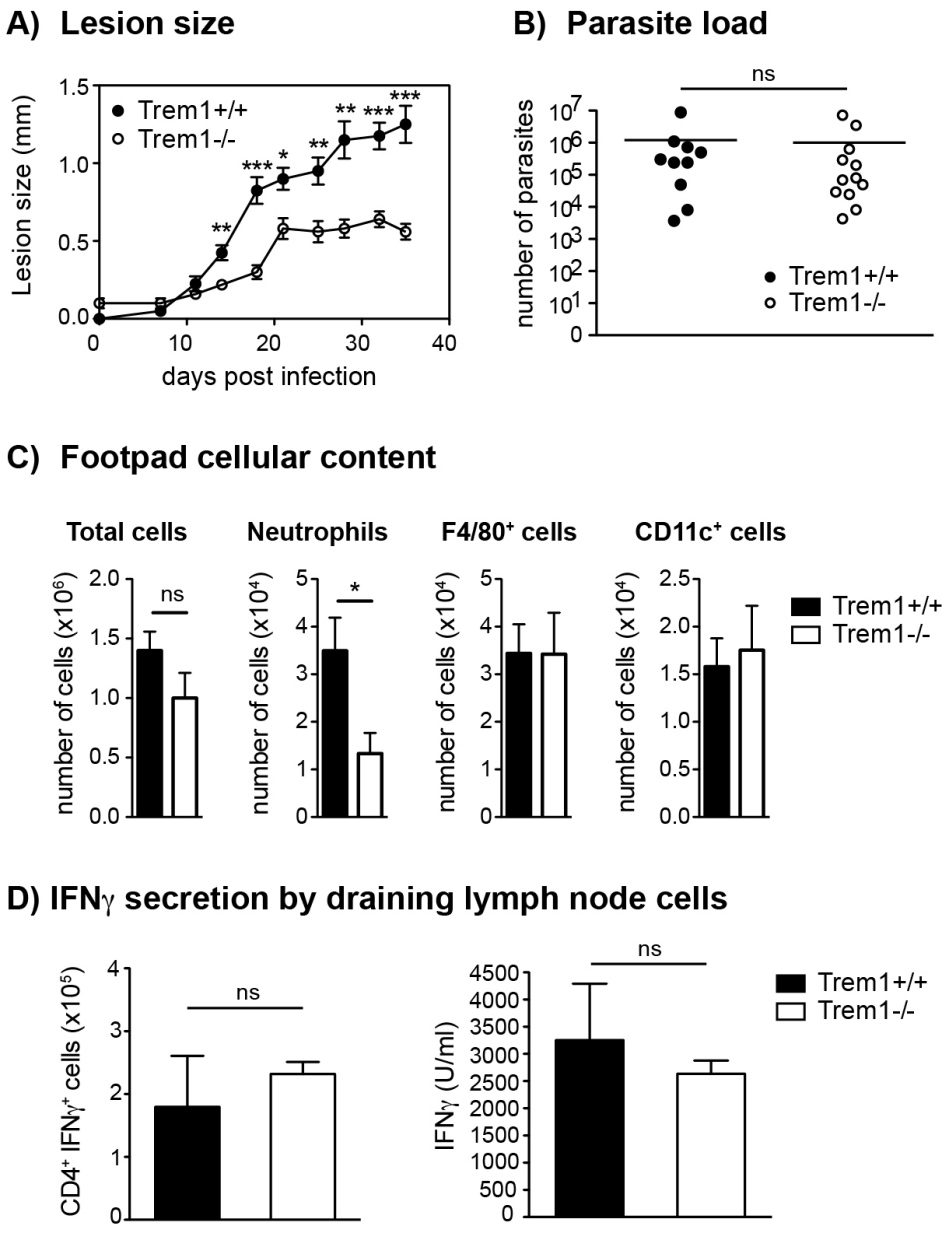
*Trem1^−/−^* mice develop smaller inflammatory lesions and show decreased cellular infiltrates at *L. major* infection sites. (A) *Trem1^+/+^* and *Trem1^−/−^* mice were inoculated with 3×10^6^ *L. major* promastigotes s.c. in the footpad and lesion development was measured over time. Each data point represents the mean lesion size ± SEM of n = 5 mice analysed per group. (B) Parasite load was assessed at 35 days post infection (p.i.) by limiting dilution analysis. (C) Infected footpads from *Trem1^+/+^* and *Trem1^−/−^* mice (n = 4–5 mice per group) were isolated 21 days p.i., digested and the cellular content was analysed by flow-cytometry. Data show mean values ± SEM and are representative of two independent experiments. (D) Draining lymph node cells from *Trem1^+/+^* and *Trem1^−/−^* mice (n = 4 mice per group) were isolated 35 days p.i.; the frequency of CD4^+^ IFNγ^+^ T cells was analysed by intracellular FACS staining or cells were re-stimulated with UV-treated *L. major* parasites and IFNγ levels in the supernatants were assessed by ELISA. Data show mean values ± SEM of triplicate measurements. Representative data from one out of three independent experiments are shown. ***, p<0.001; **, p<0.01; *, p<0.05.

Upon injection of 3×10^6^ *L. major* promastigotes s.c. in the footpad of *Trem1^+/+^* and *Trem1^−/−^* mice, an attenuation in lesion development was apparent in *Trem1^−/−^* mice already at 14 days post infection. From thereof, *Trem1^−/−^* mice showed a significantly decreased lesion size (Fig. 6A). Notably, however, parasite counts did not differ between *Trem1^−/−^* and *Trem1^+/+^* mice (Fig. 6B). We further analysed the cellular composition of infected footpads from *Trem1^−/−^* and *Trem1^+/+^* mice at 21 days post infection. While the overall cell counts were comparable, the cellular infiltrate in *Trem1^−/−^* mice exhibited ∼3-fold reduced neutrophil numbers (Fig. 6C). To look at the potential impact of the *Trem1*-deficiency on the priming of Th1 responses, expression of IFNγ was analysed in cells isolated from the draining lymph node.

The frequency of IFNγ-secreting CD4^+^ T cells was similar in *Trem1^−/−^* and *Trem1^+/+^* mice (Fig. 6D). In addition, comparable levels of IFNγ were detected in cells of both groups of mice upon re-stimulation *in vitro* with the parasitic antigen (Fig. 6D). These data are in line with comparable parasite killing observed in *Trem1^−/−^* and *Trem1^+/+^* mice. Thus, in the *L. major* infection model, absence of *Trem1* does not appear to have adverse consequences on the priming of adaptive immune responses and on parasite control while neutrophil-mediated inflammatory lesion development is substantially reduced.

### Stimulation *via* TREM-1 induces TNF secretion and resistance to apoptosis in SCF-^cond^Hoxb8 progenitor-derived neutrophils

**Figure 7:**
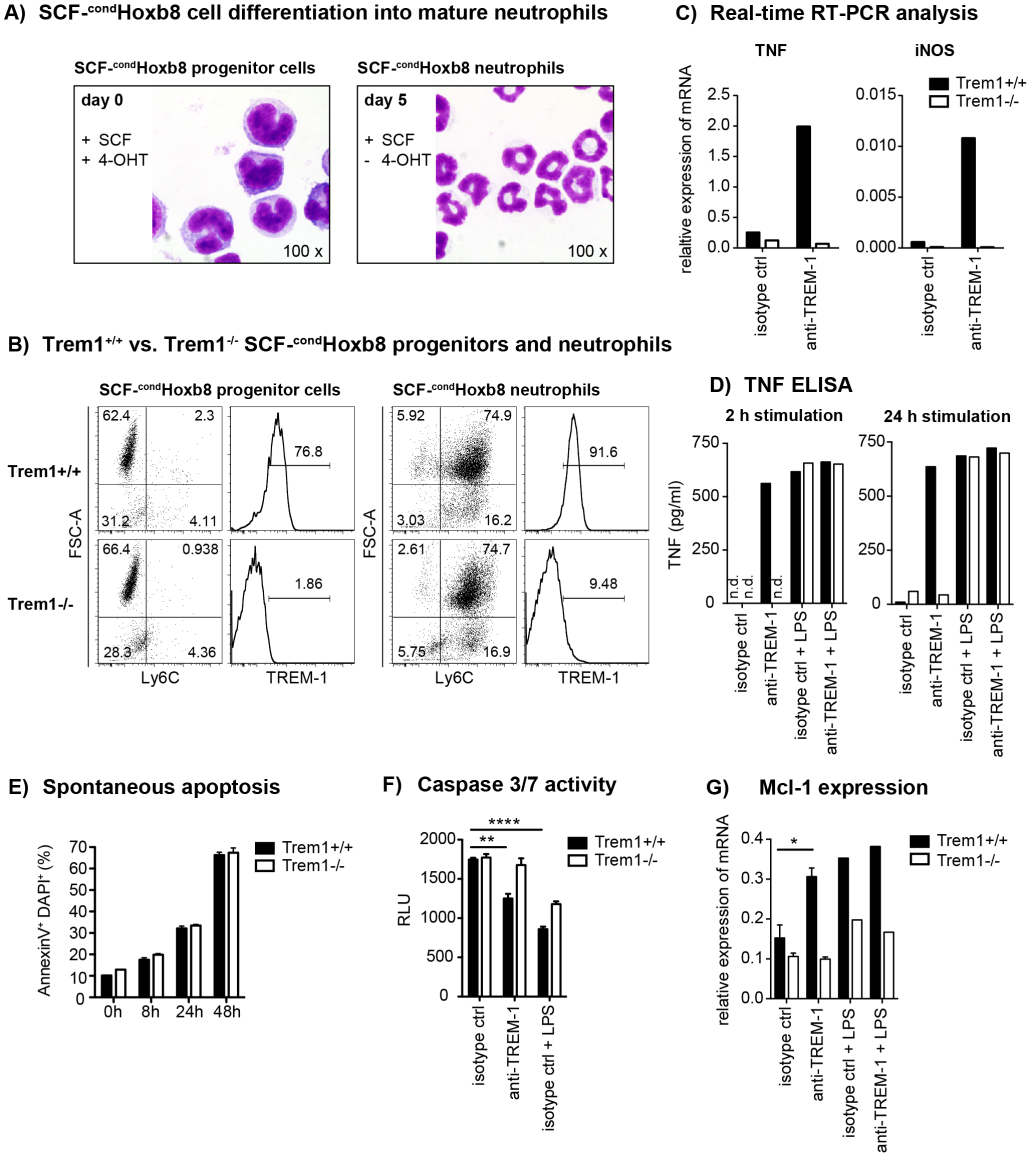
TREM-1 mediates TNF secretion and resistance to apoptosis in SCF-^cond^Hoxb8 progenitor-derived neutrophils. SCF-^cond^Hoxb8 *Trem1^+/+^* and *Trem1^−/−^* progenitor lines were generated by lentiviral transduction of Hoxb8 into BM-derived hematopoietic cells obtained from the respective mice and cultured in the presence of SCF and 4-hydroxytamoxifen (4-OHT). For *in vitro* neutrophil differentiation, SCF-^cond^ Hoxb8 cells were cultured for additional 5–6 days in the absence of 4-OHT. (A) H&E stained cytospins of *Trem1^+/+^* SCF-^cond^Hoxb8 progenitor cells (left) and differentiated neutrophils (right). (B) FACS-based characterization of *Trem1^+/+^* (top panels) and *Trem1^−/−^* (bottom panels) SCF-^cond^Hoxb8 progenitor cells (left) and differentiated neutrophils (right). (C) TNF and iNOS mRNA expression by *Trem1^+/+^* and *Trem1^−/−^* SCF-^cond^Hoxb8 differentiated neutrophils following 2 h stimulation with plate-bound agonistic anti-TREM-1 mAb or an isotype control antibody was determined by qRT-PCR. (D) TNF secretion by *Trem1^+/+^* and *Trem1^−/−^* SCF-^cond^Hoxb8 differentiated neutrophils in response to stimulation with an agonistic anti-TREM-1 mAb or an irrelevant isotype control mAb in the presence or absence of LPS (100 ng/ml) was assessed by ELISA. (E) Spontaneous apoptosis of *Trem1^+/+^* and *Trem1^−/−^* SCF-^cond^Hoxb8 neutrophils *in vitro* was analysed at 5 days post differentiation with SCF (0 h) and the indicated time-points beyond by FACS-based determination of AnnexinV and DAPI double-positive cells. Bars show mean values ± SEM for n = 3 *in vitro* replicates from one representative experiment out of three independent experiments. (F) Caspase 3/7 activity was assessed upon 24 h stimulation of differentiated *Trem1^+/+^* and *Trem1^−/−^* SCF-^cond^Hoxb8 neutrophils with plate-bound agonistic anti-TREM-1 mAb, an isotype control antibody, or LPS (100 ng/ml) as a positive survival control. Bars show mean values ± SEM for n = 3 *in vitro* replicates from one representative experiment out of three independent experiments. n.d., not detected. (G) Expression of Mcl-1 mRNA was assessed by qRT-PCR 2 h post stimulation with plate-bound anti-TREM-1 or an isotype control mAb in the presence or absence of LPS. Bars show mean values ± SEM of three independent experiments for anti-TREM-1 vs. isotype control mAb stimulated cells. n.d., not detected. ****, p<0.01; **, p<0.01; *, p<0.05.

The reduced neutrophil numbers at *L. major*-infected sites and the decreased lesion size in *Trem1^−/−^* mice agree with the notion that neutrophils play a central role in inflammatory lesion development. Indeed, the presence of non-healing lesions in *L. major* susceptible BALB/c strains is associated with elevated numbers of neutrophils [43]. Since neutrophils constitutively express high levels of TREM-1 (Fig. 1B, 4D), we aimed to investigate the consequences of TREM-1-mediated stimulation on their functional responses in more detail. Analysis of mouse neutrophils has so far been complicated by the limited numbers of cells that can be retrieved from the peripheral blood, their short life span or the distinct differentiation stages of BM-derived neutrophils. Hence, we made use of a recently described system by which neutrophils can be differentiated *ex vivo* in large numbers using conditional Hoxb8 [44]. Using a slightly modified protocol, BM-derived progenitors were lentivirally transduced with conditional Hoxb8 in the presence of SCF, resulting in immortalized myeloid progenitor lines, termed SCF-^cond^Hoxb8. Upon shutdown of Hoxb8 expression by withdrawal of 4-OHT, cells differentiate into mature neutrophils in the presence of SCF. As shown in Figure 7A and 7B, withdrawal of 4-OHT in fact induced the appearance of cells bearing the characteristic phenotype of mouse neutrophils after *in vitro* differentiation for 5 days. Moreover, *Trem1^+/+^* SCF-^cond^Hoxb8-derived mature neutrophils also expressed distinct levels of surface TREM-1 (Fig. 7B). Stimulation of *Trem1^+/+^*, but not of *Trem1^−/−^*, SCF-^cond^Hoxb8-derived mature neutrophils with a plate-bound agonistic anti-TREM-1 antibody resulted in pronounced mRNA expression of iNOS and TNF (Fig. 7C) and induced rapid secretion of TNF at levels comparable to those triggered by LPS (Fig. 7D).

Since neutrophil survival versus apoptosis could represent a deciding factor in the control of inflammation not only in the *L. major* infection model but also in the pathogenesis of experimental colitis [45], we compared the susceptibility of *Trem1^+/+^* and *Trem1^−/−^* neutrophils to spontaneous apoptosis. Following prolonged culture of fully differentiated SCF-^cond^Hoxb8-derived neutrophils *in vitro*, an increasing and comparable frequency of AnnexinV^+^DAPI^+^ cells was detected for *Trem1^+/+^* and *Trem1^−/−^* neutrophils (Fig. 7E). However, in the presence of TREM-1-mediated stimulation a reduced apoptosis rate based on diminished Caspase 3/7 activity could be observed for *Trem1^+/+^* neutrophils (Fig. 7F). Furthermore, agonistic anti-TREM-1 stimulation of *Trem1^+/+^* but not *Trem1^−/−^* neutrophils resulted in ∼2-fold upregulated mRNA expression of Myeloid Cell Leukemia-1 (Mcl-1) (Fig. 7G), analogously to what has recently been described for human TREM-1-stimulated monocytes [46].

Thus, TREM-1-mediated activation of mouse neutrophil appears to contribute to their survival.

### Influenza virus-infected *Trem1^−/−^* mice exhibit reduced morbidity but an equal capacity for viral clearance

**Figure 8:**
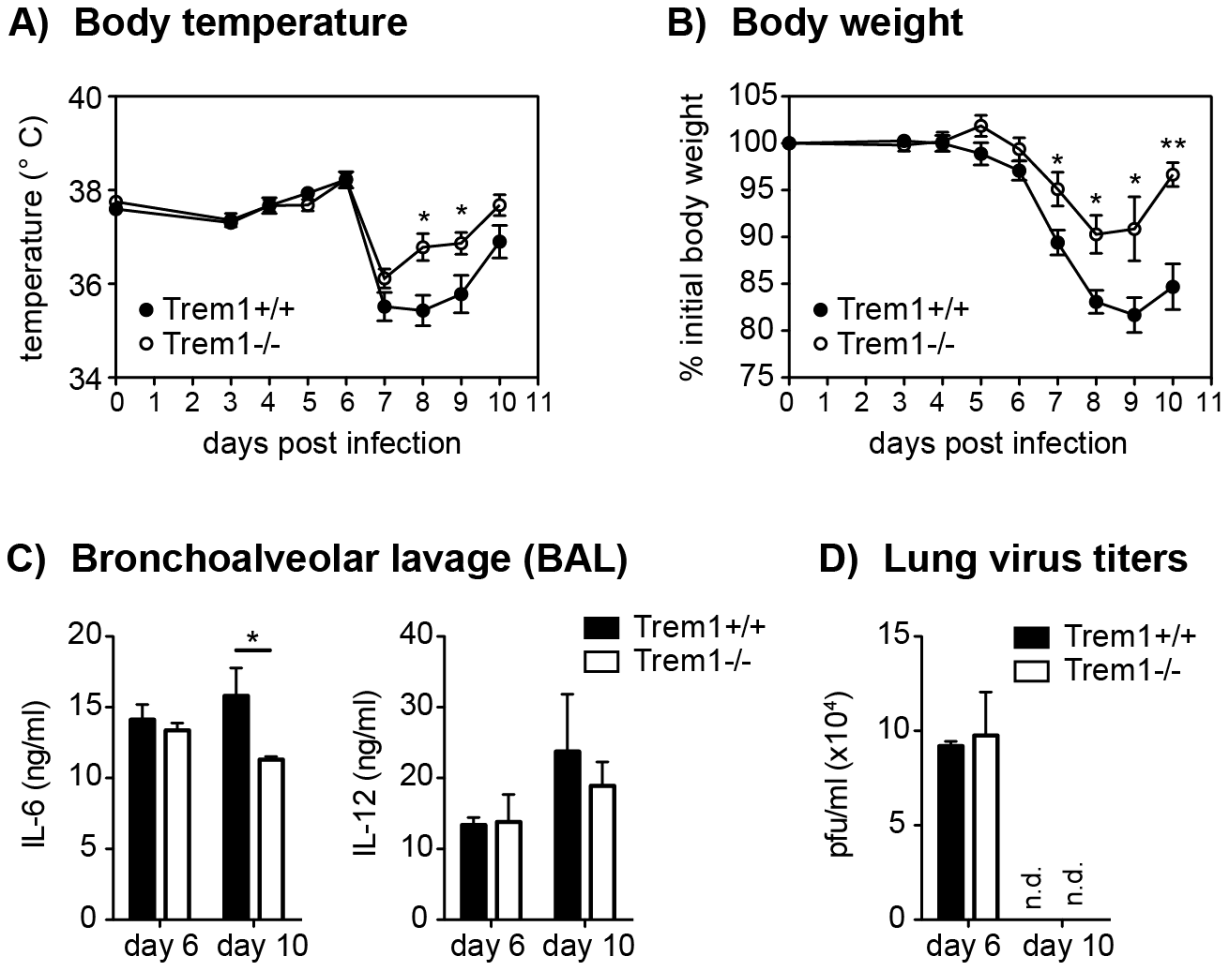
Reduced morbidity but intact viral clearance in influenza virus-infected *Trem1^−/−^* mice. *Trem1^+/+^* and *Trem1^−/−^* mice were infected intratracheally with 50 PFU influenza A virus PR8. (A, B) Body temperature and weight loss relative to the initial body weight following infection. Graphs show pooled data from two independent experiments and mean values ± SEM of n = 10 mice per group. (C, D) Mice were sacrificed at the indicated time-points post infection. Bars show mean values ± SEM of n = 4 (day 6) and n = 6 (day 10) mice per group. (C) Lung viral titers were determined by plaque assay on MDCK cells. (D) IL-6 and IL-12 levels in BAL fluid were assessed by ELISA. n.d., not detected. **, p<0.01; *, p<0.05.

Intrigued by the substantially diminished inflammatory lesions, yet intact parasite clearance in *L. major*-infected *Trem1^−/−^* mice, we aimed to substantiate these findings in an altogether different infection model. Since high expression of TREM-1 by alveolar macrophages and previously published data [28], [30], [47], [48] suggest a potential role for TREM-1 in lung inflammatory responses, we infected *Trem1^+/+^* and *Trem1^−/−^* mice intratracheally with 50 PFU influenza A virus PR8. Hypothermia and body weight loss, which are characteristically associated with experimental influenza virus infection, were observed in *Trem1^+/+^* mice at 7 days post infection (Fig. 8A and 8B). While the body temperature also dropped in *Trem1^−/−^* mice, a quicker recovery from hypothermia was seen in the *Trem1^−/−^* group (Fig. 8A). Moreover, weight loss in *Trem1^−/−^* mice was significantly attenuated and *Trem1^−/−^* mice further exhibited reduced levels of IL-6 in bronchoalveolar lavage fluid (BALF) at 10 days post infection (Fig. 8B and 8C). Notably, in spite of the reduced morbidity observed, *Trem1^−/−^* mice were equally competent in clearing the influenza virus infection as *Trem1^+/+^* controls (Fig. 8D).

### *Trem1^−/−^* mice are equally capable of clearing *L. pneumophila* as *Trem1^+/+^* controls

**Figure 9:**
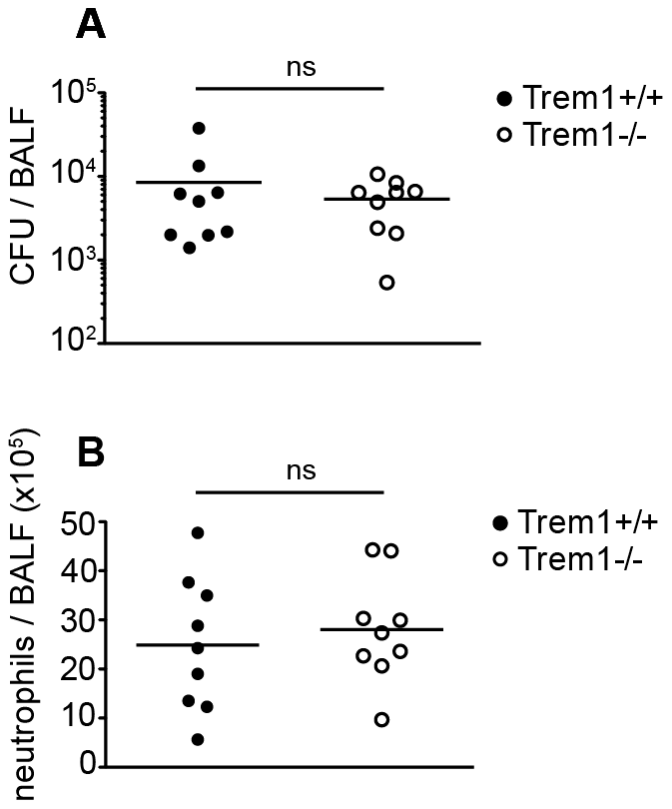
*Trem1^−/−^* mice are equally capable of clearing *L. pneumophila* as *Trem1^+/+^* controls. *Trem1^+/+^* and *Trem1^−/−^* mice were infected intranasally with 5×10^6^ CFU *L. pneumophila*. 3 days post infection CFU (A) and neutrophils (B) were quantified in the BALF. ns, no statistically significant difference. Pooled data from two independent experiments are shown.

After having established that deficiency in TREM-1 attenuates disease but does not impair pathogen control during a parasitic and viral infection, we last sought to address the significance of TREM-1 in a bacterial infection model. Indeed, controversial results have been obtained with respect to the importance of TREM-1 in microbial control following infection of experimental animals with *Pseudomonas aeruginosa* [28], [30]. Here, we employed a *Legionella pneumophila* infection model which also causes severe upper airway inflammation in permissive mice and critically depends on neutrophil-mediated control [49], [50], [51]. As shown in Figure 9, at 3 days post infection with 5×10^6^ CFU of *L. pneumophila*, CFU and neutrophil numbers in the BALF did not significantly differ between *Trem1^+/+^* and *Trem1^−/−^* mice. Furthermore, at day 5 post infection CFU were reduced to < 500/BALF in both groups of mice with no significant differences observed between *Trem1^+/+^* and *Trem1^−/−^* mice (data not shown).

## Discussion

The significance of TREM-1 as a central amplifier of acute pro-inflammatory responses during endotoxin-induced shock and microbial sepsis is well established. However, increasing evidence, including the recently reported association of TREM-1 with the DAMP protein HMGB1 [26], [27], also suggests a potential role for TREM-1 during non-infectious and chronic inflammatory conditions. In line with this notion, we have previously described a crucial involvement of TREM-1 in IBD as based on the significant amelioration of experimental colitis upon blockade of TREM-1 with the antagonistic LP17 peptide [22]. Blocking TREM-1 signaling by daily administration of TREM-1-Ig fusion proteins or synthetic analogues in chronic disease models is not only costly and straining but also fails to cover for the possibility that the yet unidentified TREM-1 ligand may signal through alternative receptors.

Here, we have generated a *Trem1^−/−^* mouse to unambiguously investigate the impact of a complete TREM-1-deficiency on the pathogenesis of experimental colitis but also of two other distinct sub-acute disease settings where the role of TREM-1 has so far not been addressed *in vivo*, i.e. inflammation induced by a parasitic and viral infection. Our findings demonstrate that *Trem1^−/−^* mice not only show a highly attenuated CD4^+^ T cell- and DSS-induced colitis but also display significantly reduced lesion size and diminished morbidity during infections with *L. major* and influenza virus, respectively.

The substantial attenuation of illness and immune-mediated pathologies in *Trem1^−/−^* mice across these distinct models suggests a common mechanism by which TREM-1 signaling promotes inflammation irrespective of the original trigger. Several non-exclusive scenarios can be considered that may account for the attenuated disease in *Trem1^−/−^* mice: *A priori* reduced chemotactic recruitment of *Trem1^−/−^* neutrophils and monocytes, diminished pro-inflammatory activities, and/or reduced life-span of myeloid cell subsets. Although we observed significantly decreased numbers of distinct myeloid cell subsets in the LP of colitic *Trem1^−/−^ x Rag2^−/−^* mice and at *L. major*-infected sites in *Trem1^−/−^* mice, we consider it unlikely that deficiency in TREM-1 causes an intrinsic primary defect in chemotaxis. When we analysed *L. major* infected sites at an early time-point, i.e. 3 days post infection, no differences in cellularity were detected between *Trem1^+/+^* and *Trem1^−/−^* mice (data not shown). Moreover, a recent study clearly demonstrated that TREM-1/3 proteins are not required for transendothelial migration of neutrophils [30]. Nonetheless, the markedly decreased expression of mRNA for monocyte- (CCL2), granulocyte- (CXCL1, CXCL2, CXCL5) but also T cell (CXCL9) chemoattractants in the LP of *Trem1^−/−^ x Rag2^−/−^* mice will in a secondary manner certainly have contributed to the decreased accumulation of inflammatory cells. Besides the diminished expression of chemotactic mediators, the colonic LP of colitic *Trem1^−/−^ x Rag2^−/−^* mice also exhibited substantially reduced mRNA levels of several innate cytokines, including IL-1β, IL-6 and TNF. The reduced expression of pro-inflammatory mediators in the entire colonic LP in *Trem1^−/−^ x Rag2^−/−^* mice at the late stage of colitis induction certainly also mirrors the decreased cellular infiltration. However, considering the capacity of TREM-1 to augment the production of several chemotactic mediators either directly in infiltrating myeloid cells or likely also indirectly, in a paracrine manner (e.g. through secretion of IL-1β) [4], [22], [52], we believe that TREM-1-amplified production of pro-inflammatory cytokines represents a key early pathogenic event that will ultimately determine the later disease course and may largely account for the attenuated disease in *Trem1^−/−^* mice. In this respect, it is noteworthy that the colonic LP of colitic *Trem1^−/−^* mice contained markedly fewer MHCII^int^ Ly6C^hi^ cells or inflammatory macrophages with the capacity for expression of pro-inflammatory mediators [35], [36].

As we have employed a CD4^+^ T cell-dependent colitis model and indeed observed considerably reduced CD4^+^ T cell numbers and correspondingly also mRNA levels for IFNγ and IL-17 in the colonic LP of transferred *Trem1^−/−^ x Rag2^−/−^* mice, the question arises whether deficiency in TREM-1 may directly impact the priming of adaptive immune responses. Whereas in the colitis model we have not analysed CD4^+^ T cell responses in more detail, CD4^+^ T cells isolated from *L. major*-infected and CD8^+^ T cells retrieved from influenza virus-infected *Trem1^−/−^* mice, respectively, exhibited an unimpaired capacity for IFNγ production compared to T cells from *Trem1^+/+^* mice.

The deciding role of neutrophils in the *L. major* infection model [42] and the substantially decreased lesion size in *Trem1^−/−^* mice have prompted us to investigate the impact of TREM-1 on neutrophil-mediated functions in more detail. In particular, we were interested in the potential modulatory effect of TREM-1 ligation on neutrophil survival as delayed neutrophil apoptosis could also represent a critical pathogenic factor in intestinal inflammation [45]. In agreement with a previous report [30], deficiency in TREM-1 caused no intrinsic predisposition for increased spontaneous neutrophil apoptosis. However, agonistic TREM-1 stimulation significantly promoted survival of SCF-^cond^Hoxb8 progenitor-derived neutrophils *in vitro*. We have also sought to assess the frequency of apoptotic neutrophils in *Trem1^+/+^* versus *Trem1^−/−^* mice *in situ* by staining colonic tissue sections from colitic mice for cleaved caspase 3 (data not shown). However, due to the likely very rapid clearance of apoptotic neutrophils by intestinal phagocytes, cleaved caspase 3 positive cells were rare in *Trem1^+/+^* mice and even more scarce in *Trem1^−/−^* mice. Moreover, the substantially decreased cellular infiltrate in *Trem1^−/−^* mice further precluded an objective comparison and quantification of apoptotic cells *in situ*. While we cannot present definitive evidence that TREM-1 prolongs neutrophil survival *in vivo*, we still believe that the reduced presence of neutrophils in *Trem1*-deficient mice in the CD4 T cell induced colitis and *L. major* infection model relates to both: A reduced secondary recruitment based on the diminished expression of neutrophil chemotactic mediators and to a reduced life-span in the absence of TREM-1 ligation, with the former mechanism numerically perhaps being more relevant.

Considering the various effector cell types and mechanisms by which TREM-1-mediated stimulation could contribute to disease, it may appear intriguing that deficiency in TREM-1 did not completely protect from disease. Accordingly, the degree of protection from colitis was not much higher in *Trem1^−/−^* mice compared to mice that were treated with the antagonistic LP17 peptide in our previous study [22]. We hypothesize that the absence of complete protection in *Trem1^−/−^* mice may primarily relate to the role of TREM-1 as an amplifier but not inducer of pro-inflammatory reactions [3], [5]. While we cannot rule out a potential participation of TREM-3, we believe that the protective effects seen in *Trem1^−/−^* mice are too substantial for a major involvement of TREM-3 in the inflammatory models analysed.

One of the most striking findings of the present study was the observation that microbial control in the models analysed was apparently not impaired in *Trem1^−/−^* mice in spite of the blunted inflammatory responses. Hence, while *Tnf^−/−^* or anti-TNF-treated mice exhibit an aggrevated acute DSS-induced colitis [39], [40] and also show enhanced parasite and bacterial burdens upon infection with *L. major* and *L. pneumophila*, respectively [41], [53], *Trem1^−/−^* mice appeared equally capable of controlling a parasitic, viral and bacterial infection as *Trem1^+/+^* controls. This observation is in line with the main function of TREM-1 as an inflammatory fine-tuner which still allows for pro-inflammatory TLR or NOD-like receptor-induced reactions in its absence. Moreover, TREM-1 does not appear to be involved in phagocytic or direct antimicrobial activity of myeloid cells [30], [54], [55]. Still, conflicting data on the effect of a TREM-1 blockade on microbial control have been reported from various bacterial challenge models. Injection of a TREM-1/IgG fusion protein allowed for sufficient control of an *E. coli*-induced peritoneal infection and conferred protection [9] whereas maximal but not half-dose siRNA silencing of TREM-1 increased mortality in a fecal peritonitis model [29]. Similarly, administration of the antagonistic LP17 peptide protected rats from a *P. aeruginosa*-induced pneumonia [28], whereas complete deficiency in TREM-1/3 led to markedly increased mortality in *Pseudomonas aeruginosa*-challenged mice due to defective transepithelial migration of neutrophils [30]. It has been proposed that the degree of TREM-1 blockade was a likely critical parameter to account for these disparant findings [29], [30]. However, our findings demonstrate that microbial control must not necessarily be impaired in mice with a complete deficiency in TREM-1, even when employing a *L. pneumophila* infection model where early neutrophil accumulation is also crucial for bacterial clearance [49], [50], [51]. Thus, we speculate that analogous to the divergent results obtained for endotoxin-challenged DAP12-deficient mice [56], [57], possibly the infection dose, the nature of the microbial agent and/or the kinetics of the infection may be critical parameters regarding the requirement for TREM-1. Accordingly, in the presence of low abundance and/or low affinity TLR ligands or fast replicating agents, the inflammatory response may more stringently depend on TREM-1-amplified signaling. Alternatively, the expression of TREM-1 and its association with DAP12 may only preferentially be induced in situations of high abundance and/or high affinity TLR ligands whereas low level signaling would favour the engagement of TREM-2.

In summary, while the impact of TREM-1 on microbial control still needs further investigations across different experimental models, our extensive characterisation of *Trem1^−/−^* mice shows an unanticipated prominent role for TREM-1 in parasitic and viral infections. Our findings thus suggest that therapeutic targeting of TREM-1 holds considerable promise for distinct non-infectious and infectious inflammatory disorders and may bypass the increased risk for impaired microbial control which is associated with the general targeting of TNF.

## Materials and Methods

### Mice

Breedings and cohort maintenance were performed under SPF conditions in isolated ventilated cages in the central animal facility of the Medical School, University of Bern. All studies were conducted with age- and sex-matched animals. *Trem1^+/+^* mice that were used as wildtype controls were originally derived from the interbreeding of *Trem1^+/−^ x Cre^+/−^* mice, thus carrying the identical C57BL/6 genetic background and representing former littermates of *Trem1^−/−^* mice. To nonetheless adjust for potential differences in the microflora composition in *Trem1^−/−^* vs. *Trem1^+/+^* mice, the bedding of *Trem1^+/+^* and *Trem1^−/−^* mice was regularly exchanged between cages three weeks prior to the start of the experiments.

### Ethics statement

All animal experiments were approved by the Veterinary Offices of the Cantons of Bern, Lausanne and Zurich and performed in compliance with Swiss laws for animal protection.

### Generation of *Trem1*-deficient mice

The generation of *Trem1*-deficient mice was designed and carried out in collaboration with the TaconicArtemis GmbH (Köln, Germany). To account for potential lethal effects of a total deletion of the *Trem1* gene and to allow for a possible cell-specific ablation of *Trem1* expression, a targeting vector was designed for conditional deletion of exon 2, which encodes the extracellular part of *Trem1* and also contains the putative ligand binding site [31]. As illustrated in the supplemental material Fig. S1, the targeting vector was constructed on the basis of the cloning vector KS loxP ftr Neo BS to flank exon 2 with loxP sites, to comprise additional restriction sites (AseI and AvaI) and to contain PuroR (flanked by F3 sites) and Neomycin (flanked by FRT sites) positive selection marker cassettes to control for homologous recombination upstream and downstream of exon 2, respectively. For counterselection, a Tk cassette was included downstream of exon 4. As a template for the PCR reactions a BAC-based plasmid containing the entire genomic mouse *Trem1* locus (RP23-32N8) was obtained from BACPAC Resources Center BPRC (Oakland, USA). The targeting vector was electroporated into a C57BL/6N.tac embryonic stem cell line (TaconicArtemis). On day 2, cells were selected with Puromycin and G418 and on day 8 counterselection with Gancyclovir was initiated. Isolated and expanded ES clones were screened for complete integration of the targeted allele by standard Southern blotting analyses with probes located upstream of exon 1 (5e2) or exon 3 (ila1) (Fig. S1). Primer sequences for generation of the 5e2 Southern probes were: CGGATTTGACCAGGAATGACAG(sense) and CTTCCAGTTCATTCATGGACAGC (antisense) and for the ila1 Southern probe: AGCTCCTCTTGTCTGCCATTCAAGGC (sense) and GGCTACAACCTTGTTCTGCAG (antisense). Eight positive clones could be identified and the ES clone A-A5 was subsequently injected into Balb/c derived blastocytes which were then transferred to pseudopregnant NMRI females. Chimeric offspring were bred to C57BL/6 female mice *(C57BL/6-Tg(ACTB-Flpe)2Arte*, TaconicArtemis) transgenic for *Flp* recombinase to achieve deletion of the FRT and F3 flanked selection cassettes PuroR and Neomycin, respectively. Germline transmission of the targeted *Trem1* allele was identified by coat color contribution and by PCR using oligo 1_sense (GTGCTCAGAGAATGTCTTTGTATCC) and oligo 4_antisense (CCCTGGTCAGACCATTTACC) which either yield a 1.3 kb fragment for the wildtype (WT) allele or a 1.6 kb fragment for the conditional *Trem1^flox^* allele. (Fig. S1). Cycling conditions were: 5′ at 95°C followed by 35 cycles consisting of 30″ at 95°C, 30″ at 60°C, 1′ at 72°C, followed by 10′ at 72°C. Thus identified *Trem1^+/flox^* mice (*C57BL/6-TREM-^1tm1821_33.1Arte^*) were mated with male mice carrying the Cre recombinase under the control of the ROSA26 locus (C57BL/6-Gt(ROSA)26Sor^tm16(Cre)arte^, TaconicArtemis) to obtain systemic deletion of one *Trem1* allele (*Trem1^+/−^*). *Trem1^+/−^ x Cre^+/−^* mice were interbred to achieve deletion of *Cre* and to obtain wildtype controls (*Trem1^+/+^*) and heterozygous (*Trem1^+/−^*) and homozygous (*Trem1^−/−^*) *Trem1*-deficient mice. Deletion of exon 2 in *Trem1^+/−^* and *Trem1^−/−^* mice was assessed by the genotyping PCR strategy described above and depicted in Fig. S1. *Trem1^+/+^* and *Trem1^−/−^* mice were subsequently expanded for experiments. For the CD4 adoptive transfer model of colitis, *Trem1^−/−^ x Rag2^−/−^* mice were generated by crossing *Trem1^−/−^* mice with Helicobacter^+^ *Rag2^−/−^* mice and interbreeding of the F1 offspring. The Helicobacter^+^ status of the *Trem-1^−/−^ x Rag2^−/−^* offspring was confirmed by PCR testing (Microbios GmbH, Reinach, Switzerland).

### Flow cytometry (FACS)

The following mAbs were used: anti-mouse CD11b-Pacific Blue (M1/70), CD45-Pacific Blue (30-F11), CD45-Brilliant Violet570 (30-F11), CD4-APC-Cy7 (RM4-5), Gr1-PE (RB6-8C5), NK1.1-PE-Cy7 (PK136), Ly6G-APC-Cy7 (1A8) and F4/80-biotin (BM8) IL-7Rα-biotin (A7R34), CD3ε-biotin (145-2C11) CD19-biotin (6D11), Gr1-biotin (RB6-8C5) and Ter119-biotin (TER119), all purchased from Biolegend; anti-mouse CD115-PE (AFS98), Gr1-APC (RB6-8C5), CD45-eFluor605 (30-F11), CD45.1-PE (A20), MHCII-APC (M5/114.15.2), CD11c-PE (N418), CD11b-eFluor450 (M1/70), Streptavidin-PE-Cy7, were purchased from eBioscience (San Diego, USA); anti-mouse Ly6C-FITC (AL-21) from BD Pharmingen (San Diego, USA) and anti-mouse TREM-1-APC (174031) from R&D Systems. DAPI (Invitrogen) was used in a final concentration of 0.5 µg/ml to exclude dead cells. Prior to FACS stainings, Fc receptors were blocked using supernatant from the hybridoma 2.4G2. Cells were acquired on a LSRII SORP (BD Biosciences, San Diego, USA) and analysed using FlowJo cytometric analysis program (Tree Star, Ashland, USA).

### Impact of TREM-1 on hematopoiesis

#### Analysis of hematopoietic stem cells and progenitors

For FACS analysis of stem cell enriched LSK cells and myeloid progenitors, and also for determination of colony-forming units, a prior lineage depletion was performed using biotinylated mAbs against red blood cell precursors (α-Ter119), B cells (α-CD19), T cells (α-CD3ε), myeloid cells (α-Gr1), MACS α-biotin beads, and LS columns (Miltenyi Biotec). Lymphoid progenitors were further removed by adding anti-IL-7Rα-biotin.

#### Determination of colony forming units

3.33×10^3^ lin^−^ cells were transferred into methocult base medium (M3134; Stemcell Technologies) supplemented with 15% FCS, 20% BIT (50 mg/ml BSA in IMDM, 1.44 U/ml recombinant-human (rh) insulin (Actrapid, Novo Nordisk) and 250 ng/ml human holo transferrin [Prospec]), 100 µM 2-β-mercaptoethanol, 2 mM l-glutamine, penicillin/streptomycin, and 50 ng/ml recombinant-mouse SCF (rmSCF), 10 ng/ml rm–IL-3, 10 ng/ml rh-IL-6 and 50 ng/ml rm-Flt3-ligand (all from Prospec). Colonies and cells were enumerated after 7 days (≥30 cells/colony).

#### Generation of mixed bone marrow chimeras

Congenic (CD45.1^+^) recipient mice were irradiated in two split doses with 650 cGy in a 4 h interval in a Gammacell 40 exactor (Best Theratronics). Total donor bone marrow (BM) was collected from *Trem1^−/−^*, *Trem1^+/+^* and *Trem1^+/+^ x GFP^+/+^* mice as described below. Donor BM was mixed 1∶1 and 15×10^6^ total cells of either mixed *Trem1^−/−^* and *Trem1^+/+^ x GFP^+/+^* BM cells or mixed *Trem1^+/+^* and *Trem1^+/+^ x GFP^+/+^* BM cells were transferred in 200 µl PBS i.v. into the irradiated recipient mice. After transfer, the recipients were treated with antibiotics (Baitryl and Bactrim) in the drinking water for two weeks. After 10 weeks, the grade of chimerism was controlled in the peripheral blood by calculating the CD45.2^+^/CD45.1^+^ ratio. In all chimeras, the grade of chimerism among the circulating myeloid cells was at least 99%.

### Generation and analysis of SCF-dependent conditional Hoxb8-immortalised progenitor cells and neutrophils

#### Generation of Hoxb8 progenitor lines and differentiation of neutrophils

The protocol was adapted from the method described by Wang et al. [44] with the major modification of using a different inducible expression system [58]. In brief, bone marrow-derived haematopoietic progenitors were isolated from *Trem1^+/+^* and *Trem1^−/−^* mice by magnetic bead-based lineage depletion (BD IMag™ mouse hematopoietic progenitor cell enrichment set-DM, BD Biosciences) following the manufacturer's instructions. 2–5×10^5^ lineage^−^ cells were incubated for 36 h in complete RPMI Medium (RPMI 1640/Glutamax supplemented with 10% FCS, 1% penicillin/streptomycin solution and 50 µM 2-mercaptoethanol). Cells were then transduced with pF-5×UAS-Hoxb8(mm)-SV40-puro-GEV16 lentiviral particles by spin-infection (1000 rpm, 2 h, 30°C) in presence of 8 µg/ml polybrene. Cells were subsequently cultured in complete RPMI medium supplemented with SCF (added as 10% of CHO/SCF(mm)-conditioned supernatant) and Hoxb8 expression was induced by addition of 100 nM 4-OHT. Transduced cells were positively selected with 1.0 µg/ml puromycin for a minimum of one month. The resulting immortalized cell lines were termed SCF-^cond^Hoxb8. For *in vitro* differentiation of SCF-^cond^Hoxb8 cells into mature neutrophils, cells were washed twice in PBS to remove all traces of 4-OHT and were then replated at 20'000 cells/ml in complete RPMI medium containing SCF (but no 4-OHT). Mature neutrophils were thus obtained after 5–6 days as judged by morphology and surface expression of CD11b and Gr-1, as well as loss of c-kit (CD117) as determined by flow cytometry.

#### Apoptosis measurements

Spontaneous apoptosis of mature *Trem1^+/+^* versus *Trem1^−/−^* neutrophils was assessed at 5 days post differentiation in SCF-containing medium and 8 h, 24 h, and 48 h later. Immediately upon removal from plates, neutrophils were washed in cold PBS and AnnexinV Binding Buffer and stained with FITC-conjugated AnnexinV (BD Pharmingen) according to the manufacturer's instructions. DAPI (Sigma-Aldrich) was added immediately prior to aquisition by flow cytometry. Only AnnexinV and DAPI double-positive cells (AnnexinV^+^ DAPI^+^) were considered in the analysis of apoptotic cells. For determination of TREM-1-mediated effects on apoptosis induction, 5 days differentiated neutrophils were stimulated *in vitro* in 96-well U-bottom plates at 2×10^5^ cells/well in complete RPMI medium lacking SCF in the presence of 10 µg/ml plate-bound anti-TREM-1 mAb (MAB1187; R&D) or a respective isotype control (RTK2758, Biolegend) for 24 h. Since the relative stickiness of neutrophils activated by plate-bound anti-TREM-1 did not allow for removal from the plates and FACS-based analysis of AnnexinV^+^ DAPI^+^ cells without causing a substantial bias by the selective analysis of non-adherent cells, Caspase 3/7 activity was determined by the ApoTox-Glo assay (Promega) according to manufacturer's instructions.

### Experimental animal models

#### CD4 T cell adoptive transfer model of colitis

Colitis was induced in (Helicobacter-positive) *Rag2^−/−^* and *Trem1^−/−^ x Rag2^−/−^* mice by adoptive transfer of 2×10^5^ CD4^+^ CD25^−^ CD45RB^hi^ FACS-sorted T cells as described previously [59]. Mice were sacrificed at 12–13 days post CD4 T cell transfer at the onset of clinical signs of colitis (diarrhea, weight loss, symptoms of abdominal pain) in *Trem1^+/+^* mice.

#### Dextran sodium-sulfate (DSS)-induced colitis

Colitis was induced in (Helicobacter-negative) *Trem1^+/+^* and *Trem1^−/−^* mice by administration of 3% 36'000–50'000 MW DSS (MP Biomedicals, Solon, USA) in the drinking water for 5 days followed by 4 days of regular tap water and euthanization at 9 days.

#### *Leishmania major* (*L. major*) infections

*L. major* LV39 (MRHO/Sv/59/P strain) parasites were maintained *in vivo* in DBA/2J mice and further cultured *in vitro* in M199 medium supplemented with 10% FCS, 4% HEPES and 2% antibiotics (penicillin, streptomycin, neomycin). Mice were infected with 3×10^6^ parasites s.c. in the hind footpad in a final volume of 50 µl as previously described [41]. Footpad lesion size was measured with a Vernier caliper. The number of parasites in lesions were evaluated by limiting dilution analysis [60].

#### Influenza virus infections

Influenza virus strain PR8 (A/Puerto Rico/34 H1N1) was originally provided by J. Pavlovic (University of Zurich, Switzerland). For infections, mice were anaesthetized and inoculated intratracheally with 50 PFU influenza virus in 50 µl endotoxin-free PBS. For collection of bronchoalveolar lavage (BAL) fluid, lungs were flushed with 3×400 µl PBS. To determine influenza viral titers in the lungs, lungs were collected at the indicated time-points, homogenized and serially diluted with MDCK cells as previously described [61].

#### *Legionella pneumophila* infections

*L. pneumophila* strain JR32 (Philadelphia-1; sg1) (reference: PMID: 8225610) was grown for 3 days on charcoal yeast extract (CYE) agar plates at 37°C and resuspended in PBS prior to infection. Mice were anesthetized by i.p. injection of 5 µg xylazine/100 µg ketamine per gram body weight and infected intranasally with 5×10^6^ *L. pneumophila*. Three days post infection, mice were sacrificed and perfused and bronchoalveolar lavage fluid (BALF) was extracted with 1 ml PBS, 5 mM EDTA. CFU were quantified by plating serial dilutions on CYE agar plates and counted after 3 days incubation at 37°C. BALF neutrophils were quantified by flow-cytometry.

### Cell isolations

#### Spleen and peripheral blood

Blood was collected by tail vain incision or by cardiac puncture into heparinised PBS. Spleen cells were released by homogenization of spleens between the frosted ends of two glass slides into PBS containing 5% horse serum. Erythrocytes were depleted by brief incubation with ACK lysis buffer.

#### Bone marrow

Femurs and tibiae were removed and placed in ice-cold PBS. Remaining flesh was removed and bones were rinsed with sterile PBS. After opening of the bones, the BM was flushed with a 26 GA 3/8 needle into RPMI 10% FCS. Erythrocyte-depleted BM cells were either used for FACS-based characterizations with or without prior lineage depletion or for the generation of mixed BM chimeras.

#### Colonic lamina propria

Colons were opened longitudinally and cut into small pieces. The epithelium was removed by incubation in HBSS/HEPES containing 5% horse serum, 5 mM EDTA and 2 mM DTT at 37°C for 3×30 min under magnetic stirring. Lamina propria (LP) cells were obtained by subsequent digestion with 200 U/ml collagenase (Type IV; Sigma-Aldrich) and 50 U/ml DNase (Type I, grade II; Roche) for 2×45 min. The LP fraction was filtered through a 40 µM cell strainer, counted by Trypan Blue staining and further characterized by FACS.

#### Footpads

Footpads were digested using 1 mg/ml collagenase D in HBSS.

### Histopathological analysis of mouse intestinal tissue sections

To assess the presence of histopathological alterations on formalin-fixed, paraffin-embedded and hematoxylin-eosin-stained colonic tissue sections, a scoring system ranging from 0 (no alterations) to 15 (most severe signs of colitis) was established, including the following parameters: cellular infiltration (0–3), loss of goblet cells (0–3), crypt abscesses (0–3), epithelial erosions (0–1), hyperemia (0–2), thickness of the colonic mucosa (0–3). Histological scoring was performed by a pathologist (V.G.) blinded to sample identity.

### RNA extractions and quantitative RT-PCR analyses

RNA was isolated using RNA isolation reagent (Tri-Reagent, Molecular Research Center). DNA was digested using DNase I (Ambion), and cDNA was generated using High Capacity cDNA Reverse Transcription Kit (Applied Biosystems). Expression of genes was analysed using Qiagen Quantitect Primer Assays on a 7500 Real-time PCR System (AB Biosystems). The house keeping gene GAPDH was used for normalization of gene expression.

### Analysis of cytokines in supernatants and bronchoalveolar lavage (BAL)

Cell-free supernatants derived from SCF^−cond^ Hoxb8 neutrophils and BAL fluid from influenza virus infected mice were analysed by ELISA (Biolegend).

### Analysis of bone density

#### X-ray

High resolution X-ray analyses on anesthetized mice were performed with the MX-20 Faxitron (X-ray Corporation, Edimex, LePlessis, France).

#### MicroCT

For high resolution microcomputed tomography (MicroCT) tissues were fixed in 4% paraformaldehyde in PBS for 24 h and subsequently transferred to 70% EtOH for μCT analysis (MicroCT40, Scanco, Bruettisellen, Switzerland).

### Statistical analyses

All data were analysed with GraphPad Prism software using the Student's t-test or 2-way ANOVA.

## Supporting Information

Figure S1 **Generation of** ***Trem1*** **-deficient mice.** *Trem1*-deficient mice were generated as described in detail in the Materials and Methods section. In brief and as depicted in (A), a targeting vector was designed for conditional deletion of exon 2 (coding for the extracellular V-type Ig-like domain) by flanking of exon 2 with loxP sites. The targeting vector was further constructed to contain additional restriction sites (AseI and AvaI), the positive selection markers PuroR flanked by F3 sites and Neo flanked by FRT sites and the counterselection cassette Tk. The vector was electroporated into a C57BL/N.tac embryonic stem (ES) cell line. Genomic DNA of selected ES clones was subjected to enzymatic digestion with either AseI or AvaI and standard Southern blotting analyses with probes located upstream of exon 1 (5e2) or exon 3 (ila1) to identify successful recombination or presence of the correctly targeted allele, respectively. Balb/c-derived blastocytes injected with the so identified targeted ES clone A-A5 were then transferred to pseudopregnant NMRI females and chimerism in the offsprings was assessed by coat colour contribution (white/black). Chimeric offspring were bred to C57BL/6 females transgenic for Flp to achieve Flp-mediated removal of the F3 and FRT flanked PuroR and Neo selection markers, respectively. Germline transmission was identified by the presence of black C57BL/6 offspring, representing heterozygous floxed *Trem1* (*^+/flox^*) mice that possessed the conditional knockout allele following Flp recombination. *Trem1^+/flox^* female mice were mated with heterozygous Cre-transgenic (Cre^+/−^) “deleter” males to generate *Trem1^+/−^* mice with a heterozygous constitutive knockout allele. *Trem1^+/−^ x Cre^+/−^* mice were interbred to generate fully *Trem1*-deficient (*Trem1^−/−^*) mice. (A) Schematic presentation of the *Trem1* wildtype allele, the targeting vector, the targeted allele before and after Flp recombination *in vivo* and the final constitutive knockout allele after Cre recombination *in vivo*. (B) Southern blot analyses of the ES clone A-A5 demonstrating presence of the targeted allele (TA) following either digestion of genomic DNA with AseI and hybridization with 5e2 probe or digestion with AvaI and hybridization with ila1 probe. Restriction enzyme and Southern blot hybridization sites are indicated in (A). (C) A PCR-based genotyping strategy was developed to identify presence of the wildtype allele, the conditional knockout allele in *Trem1^+/flox^* mice and the knockout allele in *Trem1^+/−^* mice. PCR primer sites are indicated in (A).
(TIFF)

Figure S2 **Composition of immune compartments in** ***Trem1^+/+^*** **and** ***Trem1^−/−^*** **mice.** Peripheral blood (A), bone marrow (B) and spleen cells (C) from 16 weeks old age- and sex-matched *Trem1^+/+^* (n = 3) and *Trem1^−/−^* mice (n = 3) were characterized by FACS. Representative dot plots show the gating strategies for identification of the respective cell subsets and graphs show the mean values ± SEM of total cell counts of n = 3 mice per group.
(TIFF)
