# Supplementary figures and images for: TREM-1 Deficiency Can Attenuate Disease Severity without Affecting Pathogen Clearance
Source: PLoS Pathog. 2014 Jan 16;10(1):e1003900. doi: 10.1371/journal.ppat.1003900 (PMC3894224; doi:10.1371/journal.ppat.1003900)

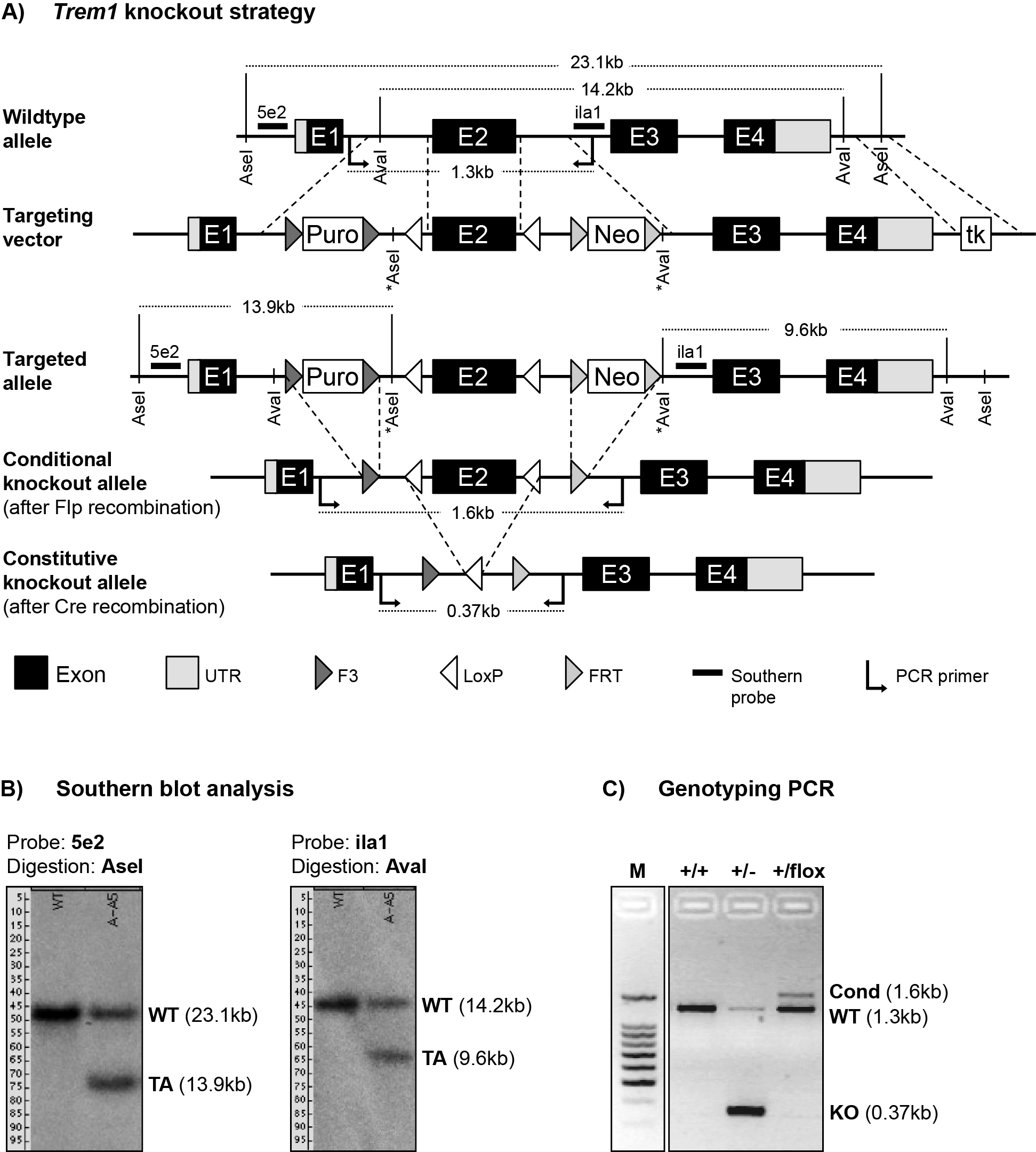

Supplement: Figure S1 — Generation of Trem1 -deficient mice. Trem1-deficient mice were generated as described in detail in the Materials and Methods section. In brief and as depicted in (A), a targeting vector was designed for conditional deletion of exon 2 (coding for the extracellular V-type Ig-like domain) by flanking of exon 2 with loxP sites. The targeting vector was further constructed to contain additional restriction sites (AseI and AvaI), the positive selection markers PuroR flanked by F3 sites and Neo flanked by FRT sites and the counterselection cassette Tk. The vector was electroporated into a C57BL/N.tac embryonic stem (ES) cell line. Genomic DNA of selected ES clones was subjected to enzymatic digestion with either AseI or AvaI and standard Southern blotting analyses with probes located upstream of exon 1 (5e2) or exon 3 (ila1) to identify successful recombination or presence of the correctly targeted allele, respectively. Balb/c-derived blastocytes injected with the so identified targeted ES clone A-A5 were then transferred to pseudopregnant NMRI females and chimerism in the offsprings was assessed by coat colour contribution (white/black). Chimeric offspring were bred to C57BL/6 females transgenic for Flp to achieve Flp-mediated removal of the F3 and FRT flanked PuroR and Neo selection markers, respectively. Germline transmission was identified by the presence of black C57BL/6 offspring, representing heterozygous floxed Trem1 (+/flox) mice that possessed the conditional knockout allele following Flp recombination. Trem1+/flox female mice were mated with heterozygous Cre-transgenic (Cre+/−) “deleter” males to generate Trem1+/− mice with a heterozygous constitutive knockout allele. Trem1+/− x Cre+/− mice were interbred to generate fully Trem1-deficient (Trem1−/−) mice. (A) Schematic presentation of the Trem1 wildtype allele, the targeting vector, the targeted allele before and after Flp recombination in vivo and the final constitutive knockout allele after Cre recombination i [file ppat.1003900.s001.tif]

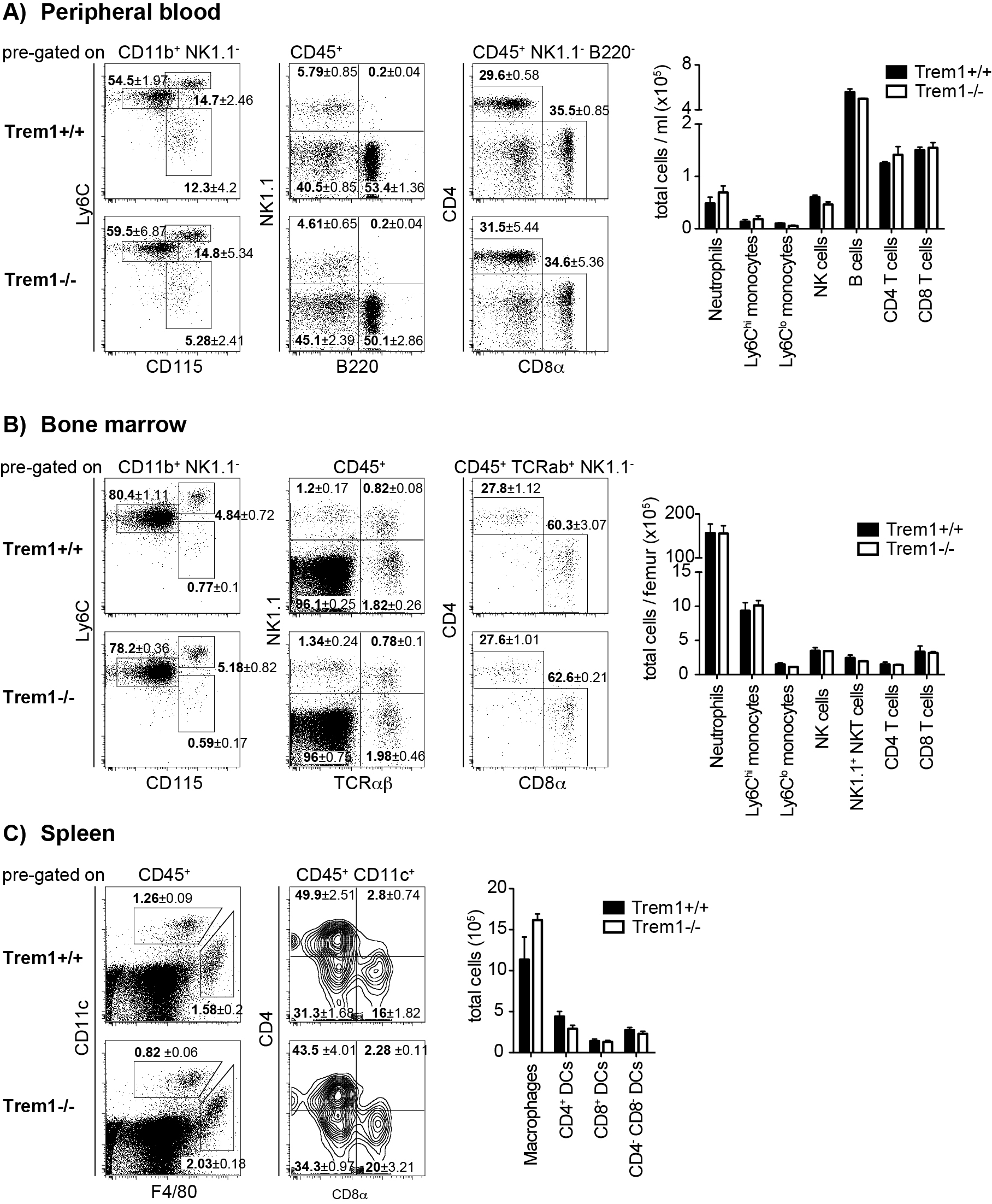

Supplement: Figure S2 — Composition of immune compartments in Trem1+/+ and Trem1−/− mice. Peripheral blood (A), bone marrow (B) and spleen cells (C) from 16 weeks old age- and sex-matched Trem1+/+ (n = 3) and Trem1−/− mice (n = 3) were characterized by FACS. Representative dot plots show the gating strategies for identification of the respective cell subsets and graphs show the mean values ± SEM of total cell counts of n = 3 mice per group. (TIFF) [file ppat.1003900.s002.tif]
